# Interpreter Communication Quality in Cognitive Assessments for Dementia: The MINDSET Randomized Clinical Trial

**DOI:** 10.1001/jamanetworkopen.2024.58069

**Published:** 2025-02-12

**Authors:** Bianca Brijnath, Simona Markusevska, Joanne Enticott, Parneet Sethi, Andrew S. Gilbert, Erika Gonzalez, Jim Hlavac, Lee-Fay Low, Dina LoGiudice, Robyn Woodward-Kron, Josefine Antoniades, Xiaoping Lin, Kerry Hwang, Jennifer White, Marina Cavuoto

**Affiliations:** 1 School of Humanities and Social Sciences, La Trobe University, Bundoora, Victoria, Australia; 2 National Ageing Research Institute, Parkville, Victoria, Australia; 3 Faculty of Medicine, Dentistry and Health Sciences, University of Melbourne, Parkville, Victoria, Australia; 4 Faculty of Medicine, Nursing and Health Sciences, Monash University, Clayton, Victoria, Australia; 5 School of Global, Urban and Social Studies, RMIT University, Melbourne, Victoria, Australia; 6 School of Languages, Literatures, Cultures and Linguistics, Monash University, Clayton, Victoria, Australia; 7 School of Health Sciences, University of Sydney, Sydney, New South Wales, Australia; 8 Department of Aged Care Medicine, Royal Melbourne Hospital, Parkville, Victoria, Australia; 9 School of Medicine and Public Health, University of Newcastle, Newcastle, New South Wales, Australia

## Abstract

**Importance:**

With increasing dementia prevalence due to population aging, interpreters are needed to facilitate timely dementia diagnosis by supporting the complex verbal and nonverbal interplay between clinicians and patients during cognitive assessments. However, to our knowledge, no randomized clinical trials have previously evaluated interventions to improve interpreter communication during cognitive assessments for dementia.

**Objective:**

To assess whether online training codesigned by interpreters, clinicians, and multilingual family carers improves the quality of interpreter communication during cognitive assessments for dementia.

**Design, Setting, and Participants:**

The Improving Interpreting for Dementia Assessments (MINDSET) study was a single-blind, parallel-group randomized clinical trial including certified interpreters and certified provisional interpreters of Arabic, Cantonese, Greek, Italian, Mandarin, or Vietnamese with at least 6 months’ experience. The trial was community based and conducted online across Australia between June 26, 2022, and April 2, 2023, with follow-up at 3 and 6 months after baseline.

**Intervention:**

Participants were randomized 1:1 to receive interpreter training during the study or, if assigned to the waiting list control, after their 6-month assessment.

**Main Outcomes and Measures:**

The primary outcome was change in the quality of interpreted communication, as measured by a weighted score comprising 5 domains: (1) knowledge of dementia, (2) cross-cultural communication, (3) briefings and debriefings, (4) interpreting skills for cognitive assessments, and (5) ethical principles relevant during a cognitive assessment. Mixed-effects generalized linear regression was conducted with random effects accounting for repeated measures from participating interpreters. Secondary analyses were conducted for differences in individual interpreting domains. A secondary per-protocol analysis included only participants who completed at least 70% of the training in the intervention group.

**Results:**

At baseline, there were 126 participants (106 [84.1%] women); mean (SD) age was 44.13 (12.71) years, mean years of interpreting experience was 8.57 (8.48) years, and 106 of 120 (88.3%) lived in an urban area. A total of 22 (17.5%) were Arabic interpreters; 14 (11.1%), Cantonese; 6 (4.8%), Greek; 14 (11.1%), Italian; 64 (50.8%), Mandarin; and 6 (4.8%), Vietnamese. The primary outcome of communication quality in the main analysis did not significantly improve in the intervention group compared with controls (mean score difference, 2.10; 95% CI, −0.43 to 4.62; *P* = .10). Per-protocol findings showed a significant intervention effect (mean score difference, 2.73; 95% CI, 0.14-5.31; *P* = .04), suggesting that the MINDSET-trained interpreters benefited in the primary outcome when they completed at least 70% of the training. Of the 5 domains, a significant intervention effect was shown in only domain 1 (knowledge of dementia) in the main analysis (mean score difference, 1.15; 95% CI, 0.54-1.77; *P* < .001) and per-protocol analysis (1.03; 95% CI, 0.27-1.79; *P* = .008) at 3 months.

**Conclusions and Relevance:**

In this randomized clinical trial, results of the primary, intention-to-treat analysis showed that interpreters’ overall interpreting communication quality did not improve in the intervention group compared with controls, but in the secondary, per-protocol analysis, it was improved when at least 70% of the training was completed. These findings suggest that an online training intervention can improve interpreters’ communication quality during cognitive assessments for dementia if at least 70% of training is completed.

**Trial Registration:**

ANZCTR Identifier: ACTRN12621001281886

## Key Points

**Question:**

Can the quality of interpreter communication in a cognitive assessment for dementia be improved with online interpreter training codesigned by interpreters, clinicians, and multilingual family carers?

**Findings:**

In this randomized clinical trial including 126 participants, communication quality in the primary, intention-to-treat analysis did not significantly improve in the intervention group compared with controls, but in a secondary, per-protocol analysis including only interpreters who completed at least 70% of training, it significantly improved.

**Meaning:**

The findings suggest that online, codesigned training can improve interpreters’ communication quality and help them mediate cognitive assessments for more timely diagnosis of dementia in multilingual populations if the interpreter has completed at least 70% of training.

## Teaser

This randomized clinical trial assesses whether interpretation training delivered online can improve the quality of interpreter communication in cognitive assessments for individuals with dementia.

## Introduction

Dementia prevalence is increasing due to population aging. Combined with global migration, more older people from ethnically diverse backgrounds will require language support from interpreters when accessing medical care, aged care, and other services.^1,2^ During cognitive assessments, interpreters play a crucial role in shaping the linguistic interplay between patients and clinicians, thus facilitating timely diagnosis, access to treatment, and postdiagnostic supports.^3,4^

Using untrained staff or family members for interpretation can result in inaccurate information being conveyed.^3,4^ Clinical guidelines recommend using interpreters who are professionally trained and certified through accredited institutions to facilitate greater accuracy, better communication, and higher satisfaction from patients and clinicians and to avoid conflicts of interest.^5,6^

However, professional interpreters may still struggle to manage the complex demands of interpreting cognitive assessments, as their generic training may not equip them with the specialist skills needed.^7^ For example, knowledge of medical terminology translations,^8^ understanding how to render disordered speech (eg, hesitations, repetition, and misused words),^9^ distinguishing between culturally vs cognitively idiosyncratic paralinguistic markers (eg, body language, facial expressions),^10^ and using nonpejorative language to describe dementia in different vernaculars^11^ are common intercultural challenges that interpreters encounter during a cognitive assessment.

Several studies have observed the influence that interpreters can have on the accuracy of cognitive assessments,^7,10,12^ and a handful have tested various training interventions to improve interpreting skills.^13,14,15,16^ However, none of these studies were randomized clinical trials, and most had small sample sizes (the largest had 61 interpreters’ complete baseline and 1-week follow-up data^16^).

The Improving Interpreting for Dementia Assessments (MINDSET) study developed a specialized online training package on dementia and cognitive assessments for interpreters. We hypothesized that compared with the control group and with their own baseline scores, interpreters who undertook the MINDSET training would show improvements in the quality of interpreter communication at 3 months after receiving the intervention.

## Methods

### Study Design

**Figure 1.**
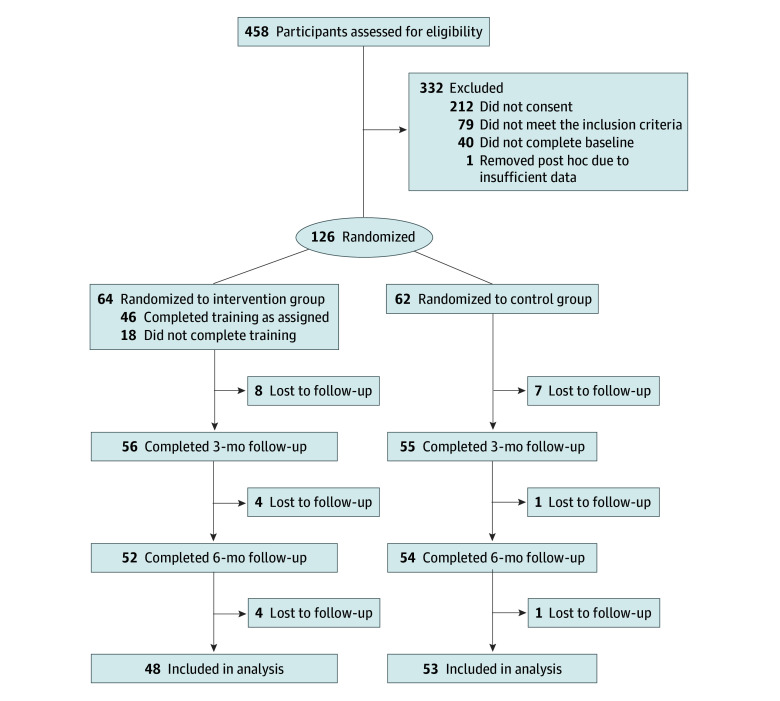
CONSORT Flow Diagram

This single-blind, parallel-group randomized clinical trial (ACTRN12621001281886) was community based and conducted online across Australia between June 26, 2022, and April 2, 2023, with assessments completed at baseline, 3 months (primary outcome), and 6 months. It was single blinded because participating interpreters randomized to the intervention group underwent the online training while the control interpreters only had access to the same training at the end of the study. The Curtin University and the University of Western Australia research ethics committees approved the trial, and the National Aging Research Institute provided governance approval. All participants provided written informed consent at enrollment. The protocol is published elsewhere^17^ and given in Supplement 1. The Consolidated Standards of Reporting Trials (CONSORT) reporting guideline was followed,^18^ and the CONSORT diagram is shown in Figure 1.

### Participants

Participants were self-identified men, women, and nonbinary persons who were interpreters certified by the Australian skills-assessing authority—the National Accreditation Authority for Translators and Interpreters (NAATI)—as either certified interpreters or certified provisional interpreters. Certified interpreters have the highest generalist interpreting accreditation in Australia and can work with complex but nonspecialized content in most situations; certified provisional interpreters are a step below certified interpreters and can work with nonspecialized community dialogue (ie, general conversation).^19^ Additionally, participants had to have at least 6 months’ experience as interpreters, internet access, and access to a computer with a webcam and microphone and had to interpret for Arabic, Cantonese, Greek, Italian, Mandarin, or Vietnamese, the most commonly spoken non-English languages of Australians aged 65 years or older.^20^ Participants were excluded if they were involved in the codesign or user testing of the MINDSET training.^21^

### Randomization and Masking

After the baseline evaluation, participants were randomized in a 1:1 ratio to receive the training during the study or, if assigned to the waiting list control, after their 6-month assessment. Assignment was based on an allocation sequence independently generated by an independent statistician through dynamic randomization using minimization.^22^ The statistician conducting randomization was blinded to participants’ baseline assessment scores. Outcome assessors were blinded to participants’ allocation.

### Procedures

Baseline assessments were completed prior to randomization. Training and assessment were completed through an online platform (assessmentQ^23^). Participants could access the platform via the internet on their own personal computers. The intervention group was given access to the training immediately after randomization and asked to complete it within 3 months, prior to undertaking their second assessment. The training was approximately 4 hours in duration and self-paced. To promote retention, participants were provided certificates following completion of all 3 trial assessments, which could be used toward Continuing Professional Development (CPD) points for mandatory NAATI recertification requirements. Participants in the intervention group were eligible for additional CPD points after completing the training.

### Outcomes

The primary outcome was change in the quality of interpreted communication as measured by a composite score comprising 5 interpreting domains: (1) knowledge of dementia and cognitive assessments, (2) cross-cultural communication, (3) briefings and debriefings, (4) interpreting skills for cognitive assessments, and (5) ethical principles relevant to the delivery of a cognitive assessment. eTable 1 in Supplement 2 describes each domain and the weightings given by the research team according to the domain’s importance for interpreting quality out of a total of 100%. Domains 1, 2, 3, and 5 were weighted 20%, 10%, 10%, and 10%, respectively, and domain 4, based on a simulated cognitive assessment that the participant had to interpret and video-record, was weighted 50% and was a hurdle requirement (ie, if domain 4 was missing, the entire assessment was considered missing, as prespecified in the protocol^17^).

Video-recorded simulations assessing domain 4 were graded by 12 (2 per language) qualified NAATI assessors using the NAATI Certified Provisional Interpreter Test Assessment Rubric,^24^ the Australian industry standard measure of interpreter performance. All the assessors were NAATI examiners and interpreting instructors at universities or vocational institutions that offered NAATI-endorsed interpreting programs. Before independently commencing their assessments, 2 senior assessors (E.G., J.H.) assessed 4 tests and generated a more detailed instruction sheet supporting the rubric for the other 12 raters to use. To ensure consistency between the 2 assessors for each language, consensus meetings were held after the first 4 video simulation assessments were completed for each language.

### Sample Size and Missing Data

A sample size calculation determined that 120 (60 test and 60 control) participants would be sufficient to detect a mean (SD) score difference between the control (mean score, 2.91) and intervention (mean score, 4.21) groups of 1.3 (2.5) with 80% power and α of .05 based on data from a similar study,^13^ assuming a normal distribution. To account for 25% sample attrition, we aimed to recruit 150 participants.

For domains 2, 3, and 5, if there were missing data on any items, the entire domain was considered to be missing, as determined by the expert research team (all authors). However, for the total Dementia Knowledge Assessment Scale score, a subcomponent of domain 1, the expert research team determined that a total score should be computed if the response rate was 80% or higher.

### Statistical Analysis

Statistical analyses were conducted using Stata, version 18.0 (StataCorp LLC). Statistical significance was set at 2-sided *P* < .05. Independent *t* tests and Pearson χ^2^ tests were used to assess between-group comparisons at baseline. Data are expressed as mean (standard error of the mean) or mean (95% CI) unless otherwise stated.

To evaluate our primary outcome, intention-to-treat analyses were performed using mixed-effects linear regressions, with random effects accounting for repeated measures from participating interpreters. Time points (baseline, 3 months, and 6 months) were categorical and specified as fixed. All other independent variables were prespecified as fixed effects. Individual domains were evaluated as secondary outcomes using the same analysis approach. Main analyses were conducted on nonimputed primary and secondary outcomes. Sensitivity analyses used the aforementioned imputed data.

All models were adjusted for group (intervention or control), time (baseline, 3 months, and 6 months), age, gender (men and women only, due to a small number of nonbinary participants), highest educational qualification, highest interpretation qualification, NAATI certification, location, and number of years of interpreting experience. All models were additionally adjusted for baseline values to avoid regression to the mean.^25^ Estimates of change within groups and differences between groups (ie, intervention effects) were obtained from marginal means and pairwise comparisons of marginal means. The moderation effect was then investigated through the interaction term for rurality, educational attainment (highest educational qualification and highest interpreting qualification), years of interpreting experience, and NAATI credential status, with group and time for the primary outcome based on the fully adjusted model.

To evaluate the sensitivity of the main findings to missing data, multiple imputation and per-protocol analyses were performed. Multiple imputation by chained equations was done using variables associated with NAATI credentials and group. Secondary per-protocol analyses, which were conducted following unblinding of the biostatistician, allocated participants to the intervention group only if they undertook 70% or more of the training; otherwise, participants were reallocated to the control group.

## Results

Overall, 458 participants were assessed for eligibility, and 79 were ineligible. A total of 168 participants enrolled, but 40 did not complete the baseline assessment and 1 was removed from the analyses post hoc due to insufficient baseline data. At baseline, there were 126 participants (64 intervention and 62 control); mean (SD) age was 44.13 (12.71) years, and mean (SD) time of interpreting experience was 8.57 (8.48) years. A total of 19 (15.1%) were men, 106 (84.1%) were women, and 1 (0.8%) was nonbinary; 106 of 120 (88.3%) lived in an urban area. Twenty-two (17.5%) were Arabic interpreters; 14 (11.1%), Cantonese interpreters; 6 (4.8%), Greek interpreters; 14 (11.1%), Italian interpreters; 64 (50.8%), Mandarin interpreters; and 6 (4.8%), Vietnamese interpreters. A total of 69 (54.8%) were certified interpreters, and 57 (45.2%) were provisional interpreters. In all, 71 interpreters (56.3%) had a postgraduate educational qualification compared with 42 (33.3%) who had a bachelor’s degree, graduate diploma, or graduate certificate and 13 (10.3%) who were high school graduates with a certificate III-IV, diploma, or advanced diploma. Sixty participants (47.6%) had a diploma, advanced diploma, or bachelor’s degree in interpreter qualifications. Overall, there were no significant between-group differences at baseline for any demographic characteristics or any of the outcome measures (Table 1). Dropout in both groups was similar, with 8 participants (12.5%) in the intervention group and 7 participants (11.3%) in the control group leaving the study at 3 months. At 6 months, 1 participant (1.6%) from the control group and 4 participants (6.2%) from the intervention group were lost to follow-up (Figure 1). For the primary outcome, the percentage of missing data (calculated using a denominator of 378, or 3 times the sum of the control [62] and intervention [64] participants) was 16.9% (64 of 378) overall; 12.4% (47 of 378) for domain 1; 9.5% (36 of 378) for domain 2; 9.5% (36 of 378) for domain 3; 13.2% (50 of 378) for domain 4; and 11.9% (45 of 378) for domain 5 (eTable 2 in Supplement 2).

**Table 1.** Baseline Characteristics of Participants by Intervention Group

Characteristic	Participants^a^		
	Total (N = 126)	Control (n = 62)	Intervention (n = 64)
Age, mean (SD), y	44.13 (12.71)	44.42 (13.00)	43.86 (12.51)
Experience, mean (SD), y	8.57 (8.48)	8.92 (8.36)	8.23 (8.66)
Primary outcome: composite score of all modules, mean (SD)	75.55 (8.71)	76.98 (7.37)	74.2 (9.68)
Secondary outcomes, mean score (SD)			
Module 1: knowledge	15.79 (1.94)	16.03 (1.87)	15.56 (2.00)
Module 2: culture	8.78 (1.72)	8.92 (1.69)	8.64 (1.76)
Module 3: briefings and debriefings	7.04 (1.73)	7.04 (1.83)	7.04 (1.64)
Module 4: interpreting skills	35.51 (7.15)	36.24 (6.49)	34.79 (7.71)
Module 5: ethics	8.04 (1.59)	7.94 (1.68)	8.15 (1.50)
Gender			
Men	19 (15.1)	11 (17.7)	8 (12.5)
Women	106 (84.1)	51 (82.3)	55 (85.9)
Nonbinary	1 (0.8)	0	1 (1.6)
Language			
Arabic	22 (17.5)	9 (14.5)	13 (20.3)
Cantonese	14 (11.1)	7 (11.3)	7 (10.9)
Greek	6 (4.8)	4 (6.5)	2 (3.1)
Italian	14 (11.1)	8 (12.9)	6 (9.4)
Mandarin	64 (50.8)	32 (51.6)	32 (50.0)
Vietnamese	6 (4.8)	2 (3.2)	4 (6.3)
RA category, No./total No. (%)			
Rural	14/120 (11.7)	5/58 (8.6)	9/62 (14.5)
Urban	106/120 (88.3)	53/58 (91.4)	53/62 (85.5)
Highest educational qualification			
High school graduate with certificate III-IV, diploma, or advanced diploma	13 (10.3)	9 (14.5)	10 (15.6)
Bachelor degree, graduate diploma, or graduate certificate	42 (33.3)	34 (54.8)	26 (40.6)
Postgraduate degree	71 (56.3)	19 (30.6)	28 (43.8)
Highest interpreting qualification			
None	19 (15.1)	9 (14.5)	10 (15.6)
Diploma, advanced diploma, or bachelor’s degree	60 (47.6)	34 (54.8)	26 (40.6)
PG certificate, PG diploma, or master’s degree	47 (37.3)	19 (30.6)	28 (43.8)
NAATI credential status			
Certified provisional interpreter	57 (45.2)	27 (43.5)	30 (46.9)
Certified interpreter	69 (54.8)	35 (56.5)	34 (53.1)

Abbreviations: NAATI, National Accreditation Authority for Translators and Interpreters; PG, postgraduate; RA, residence area.

^a^ Data are presented as number (percentage) of participants unless otherwise indicated.

The main effects model showed that on average, the intervention group had a higher overall postintervention mean score (80.42; 95% CI, 78.69-82.15) compared with the control group (78.33; 95% CI, 76.64-80.02) after adjusting for all covariates (Table 2). There was no significant difference in mean scores between the groups in the main analysis (2.10; 95% CI, −0.43 to 4.62; *P* = .10) (Table 2), but there was a significant difference in the per-protocol analysis (2.73; 95% CI, 0.14-5.31; *P* = .04) (Table 3).

**Table 2.** Intervention and Control Group Differences After Intervention for the Primary and Secondary Outcomes From the Main Intention-to-Treat Analysis

Outcome	Overall score, mean (95% CI)		Overall difference between groups	
	Control	Intervention	Mean (95% CI)	*P* value
Primary outcome	78.33 (76.64-80.02)	80.42 (78.71-82.15)	2.10 (−0.43 to 4.62)	.10
Secondary outcomes				
Domain 1: knowledge of dementia	16.12 (15.63-16.61)	17.28 (16.93-17.63)	1.15 (0.54 to 1.77)	<.001
Domain 2: cross-cultural awareness	8.89 (8.60-9.18)	9.06 (8.77-9.35)	0.17 (−0.25 to 0.59)	.43
Domain 3: briefings and debriefings	7.51 (7.22-7.80)	7.97 (7.68-8.26)	0.45 (0.01 to 0.90)	.046
Domain 4: interpreting skills	37.63 (36.41-38.85)	37.05 (35.74-38.36)	−0.58 (−2.39 to 1.24)	.53
Domain 5: ethical conduct	8.21 (7.85-8.55)	8.21 (7.94-8.48)	−0.004 (−0.44 to 0.45)	.98

**Table 3.** Intervention and Control Group Differences After Intervention for the Primary and Secondary Outcomes From the Per-Protocol Analysis

Outcome	Overall score, mean (95% CI)		Overall difference between groups	
	Control	Intervention	Mean (95% CI)	*P* value
Primary outcome	78.21 (76.68-79.74)	80.93 (78.99-82.87)	2.73 (0.14 to 5.31)^a^	.04
Secondary outcomes				
Domain 1: knowledge of dementia	16.31 (15.90-16.72)	17.32 (16.87-17.77)	1.00 (0.39 to 1.62)^b^	.001
Domain 2: cross-cultural awareness	8.79 (8.50-9.08)	9.25 (8.92-9.58)	0.46 (0.00 to 0.92)	.048
Domain 3: briefings and debriefings	7.46 (7.21-7.71)	8.18 (7.83-8.53)	0.72 (0.27 to 1.17)	.002
Domain 4: interpreting skills	37.26 (36.08-38.44)	37.44 (35.93-38.95)	0.18 (−1.82 to 2.17)	.86
Domain 5: ethical conduct	8.18 (7.89-8.47)	8.26 (7.97-8.55)	0.09 (−0.32 to 0.49)	.67

^a^ Mean difference corresponds to a percentage-point difference of 2.73, because the denominator is 100.

^b^ Mean difference corresponds to a percentage-point difference of 5.00, because the denominator is 20.

**Figure 2.**
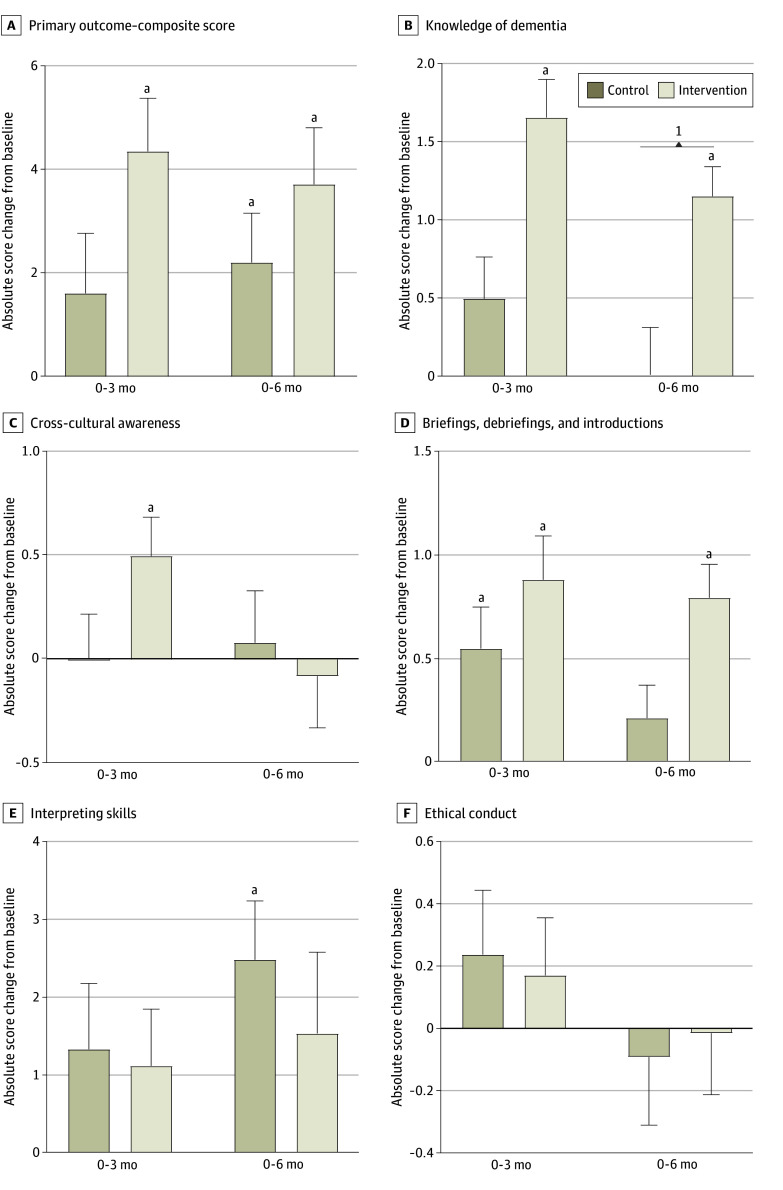
Absolute Changes in Overall Score and the 5 Domains From Baseline in the Intervention and Control Groups After 3 and 6 Months Error bars indicate SEs. ^a^*P* < .05 for within-group and between-group difference from baseline.

The analysis for the interaction between group and time showed a significant within-group score change in the intervention group at 3 months (4.34; 95% CI, 2.33-6.36) and 6 months (3.71; 95% CI, 1.57-5.85) for the primary outcome. A significant within-group change was also observed in the control group at 6 months (2.20; 95% CI, 0.34-4.07). The intervention group did not score higher compared with the control group at 3 months (between-group difference, 2.74; 95% CI, −0.43 to 5.90; *P* = .09) (Figure 2 and eTable 3 in Supplement 2). No significant intervention effects were found for the primary outcome at 6 months.

Results of the main-effects model (intention-to-treat sample) for primary and secondary outcomes after the intervention are shown in Table 2. Each of the 5 domains was examined as a secondary outcome, and there was a significant score improvement found between the intervention and control groups for domain 1 (dementia knowledge; mean score difference, 1.15; 95% CI, 0.54-1.77; *P* < .001) and domain 3 (briefings and debriefings; mean score difference, 0.45; 95% CI, 0.01-0.90; *P* = .046).

The interaction analyses supported a significant intervention effect at both 3 months (mean score difference, 1.16; 95% CI, 0.44-1.89) and 6 months (1.15; 95% CI, 0.43-1.87) for domain 1. The interaction analysis found an intervention effect at 6 months only for the briefings and debriefings domain (mean score difference, 0.58; 95% CI, 0.12-1.04). No other intervention effects were observed in other domains. Similar per-protocol findings were observed (eTables 3 and 4 in Supplement 2).

In the main effects analysis and the interaction model (group and time), NAATI-certified interpreters had higher overall mean scores by 4.83 (95% CI, 2.34-7.33) and 4.87 (95% CI, 2.38-7.38), respectively, compared with the certified provisional interpreters. No moderation effects by group or time were observed for NAATI status or rurality, educational attainment (highest educational qualification and highest interpreting qualification), and years of interpreting experience, indicating that the interaction was not significant, and the levels of these variables did not vary across groups or over time. The effect of NAATI status on the primary outcome was consistent across all groups and periods.

Imputed (sensitivity) analysis for the primary outcome showed similar findings as the main analyses and again found no improvement in the mean intervention group score (difference, 2.09; 95% CI, −0.27 to 4.46; *P* = .08) compared with the control group. The intervention effects for the dementia knowledge domain remained essentially unchanged at 3 months (mean between-group difference, 1.17; 95% CI, 0.44-1.91); this finding was similar in the nonimputed main analysis. However, a significant intervention effect for domain 3 (briefings and debriefings) was not found when examining the imputed analysis.

Of 64 intervention participants, 46 (71.9%) completed at least 70% of the training; 10 participants (15.6%) completed between 0% and 69%, and 8 (12.5%) did not attempt the training during the intervention period. Thus, 18 participants (28.1%) were reallocated to the control group for the per-protocol analysis, resulting in 46 participants in the intervention group and 80 in the control group. The intervention participants scored significantly higher on the primary outcome (mean score difference, 2.73; 95% CI, 0.14-5.31; *P* = .04) compared with the controls (Table 3). A significant intervention effect was observed at 3 months for the primary outcome (mean score difference, 3.65; 95% CI, 0.49-6.81; *P* = .02), dementia knowledge domain (mean score difference, 1.03; 95% CI, 0.27-1.79; *P* = .008), cultural awareness domain (mean score difference, 0.71; 95% CI, 0.15-1.27; *P* = .01), and briefings and debriefings domain (mean score difference, 0.73; 95% CI, 0.16-1.31; *P* = .01). These differences were maintained for the knowledge domain (mean score difference, 0.98; 95% CI, 0.31-1.65; *P* = .004) and briefings and debriefings domain (mean score difference, 0.71; 95% CI, 0.24-1.18; *P* = .003) at 6 months.

## Discussion

In this randomized clinical trial of training codesigned by interpreters, clinicians, and multilingual family carers to improve interpreters’ communication during cognitive assessments, the primary outcome in the main analysis did not significantly improve in the intervention group compared with controls. While the clinical relevance of the difference in scores was equivocal, the per-protocol findings showed a significant intervention effect, suggesting that MINDSET-trained interpreters benefited in the primary outcome when they completed 70% or more of the training. However, this was only sustained at the 3-month follow-up, suggesting that the effects of the training may be transient and that periodic reinforcement may be necessary to sustain improvements. Of the 5 domains comprising the primary outcome, domain 1 (knowledge of dementia) showed an intervention effect in the main and per-protocol analyses compared with the control group and at the 3- and 6-month follow-up. No other robust findings were observed. Therefore, the trial results are promising, but more rigorous study is required to confirm the findings.

The overall effect size for the primary outcome improvement in the per-protocol intervention sample represented a modest score improvement of 2.73 (95% CI, 0.14-5.31). The effect size for the increases in domain 1 (knowledge of dementia) also represented a modest improvement of 5.00 percentage points, as the denominator was 20 (Table 3). Modest effect sizes are expected in pragmatic interventions that are rolled out in clinical settings whereby many other uncontrolled factors can influence results.^26,27^

To our knowledge, a trial to increase quality of dementia interpretation has never been done. Several studies have highlighted the impact that interpreters can have on the accuracy of cognitive assessments,^7,10,12^ with a few using preintervention vs postintervention measures to show improvements in knowledge and confidence in interpreting skills.^13,14,15,16^ However, most studies had small sample sizes and no control group. The most promising findings were from the preintervention vs postintervention pilot study by Ono et al^13^ that included 43 interpreters (including 23 participants allocated to control) and showed that a 3-day training program for medical interpreters could facilitate improvement in patient-clinician communication.

This trial’s novelty was in its 4-hour online training codesigned by interpreters, clinicians, and multilingual family carers and specific to cognitive assessment for dementia^17^; evaluation using a digital trial format with an integrated video-simulated, interpreter-mediated cognitive assessment; and assessment of probability measures as part of the primary outcome. These aspects may explain why when compared with the control group, there was no improvement in the intervention group’s interpreter skills (domain 4). Interpreting a prerecorded video of a clinician performing a cognitive assessment of a person aged 65 years or older with discrete break points to allow for consecutive interpreting may have felt unnatural to interpreters, especially those who were less digitally savvy. Additionally, there was a practice effect that we could not control, insofar as both the intervention group and the control group improved in their interpreting skills. In-person assessments may have overcome some of these technological issues but would not have ameliorated the practice effect, would have been more invasive and expensive, and would have reduced the uniformity of the clinical simulations that we provided, especially if done with real patients (from whom consent would also need to be obtained). Thus, we used the most pragmatic approach possible to enhance feasibility and rigor in this trial.

The practical implications of this study are significant given current and predicted dementia prevalence in multilingual older people from an ethnically diverse background. In Australia, where this trial was conducted, about 40% of people aged 75 years or older speak a language other than English and have limited English proficiency.^28^ Future projections estimate a 600% or greater rise in dementia in some ethnically diverse populations.^29^ Aphasia, a common symptom of dementia, often begins with nonprimary languages (in this instance, English) and eventually progresses to primary languages.^30^ Consequently, interpreters are crucial to mediating timely diagnosis of dementia in multilingual populations, especially in countries where the population is aging and there is growing ethnic diversity among older people. Timely diagnosis can reduce known health disparities in dementia diagnosis and postdiagnostic support and help ensure that if ethnically diverse patients with dementia are diagnosed early enough, they too can benefit from the latest therapeutic advances.

### Limitations

This study has limitations. The video-simulated, interpreter-mediated cognitive assessment was challenging for some participants and may have affected results of domain 4 (interpreting skills), which was a hurdle requirement. Additionally, due to the considerable resourcing required to produce the simulated in-language assessments and rate the interpreted assessment, this trial was limited in the number of languages in which it could be conducted. Six languages were included, but there was disproportionately high representation from Mandarin-speaking interpreters, which precluded examination of results according to language groups (as per the protocol). The included languages represent the most well-established migrant cohorts in Australia, and inclusion of interpreters from emerging languages in Australia, for whom there may be fewer training opportunities, would be useful. We have extended the training across Australia to all interpreters regardless of qualification or language. A further limitation of the study was that only 71.9% of the intervention group completed the training, limiting the training’s impact across the intervention group. Interpreters were not compensated for their time in this time-intensive trial, and CPD alone may not have been enough to promote retention. As the scores for domain 2 (cultural awareness) and domain 5 (ethics) were close to ceiling at baseline, the assessment questions in these domains may not have been adequately challenging to determine a change in knowledge in these domains.

## Conclusions

In this randomized clinical trial, results of the primary, intention-to-treat analysis showed that interpreters’ overall interpreting communication quality did not improve in the intervention group compared with controls, but in the secondary, per-protocol analysis, it was improved when at least 70% of the training was completed. These findings suggest that an online training intervention can improve interpreters’ communication quality during cognitive assessments for dementia if at least 70% of training is completed. However, further studies are needed to confirm these findings and establish the most effective training duration.

## Notes

Supplement 1.
Trial Protocol

Supplement 2. **eTable 1.** MINDSET Training Program Logic **eTable 2.** Number of Participants and Outcome Measures at Each Period **eTable 3.** Primary and Secondary Outcome Changes From Baseline, Main Analysis **eTable 4.** Primary and Secondary Outcome Changes From Baseline, Per-Protocol Analysis

Supplement 3.
Data Sharing Statement
