# Supplementary material for: Interpreter Communication Quality in Cognitive Assessments for Dementia: The MINDSET Randomized Clinical Trial
Source: JAMA Netw Open. 2025 Feb 12;8(2):e2458069. doi: 10.1001/jamanetworkopen.2024.58069 (PMC11822547; doi:10.1001/jamanetworkopen.2024.58069)
Supplement: Supplement 1. — Trial Protocol [file jamanetwopen-e2458069-s001.pdf]

PROTOCOL

Improving interpreting for dementia assessments: The MINDSET Study

Protocol number: 1
Version: 1.1
Date: 10/08/2022

Authors:

A/Prof Bianca Brijnath
A/Prof Dina LoGiudice
Dr Erika Gonzalez Garcia
A/Prof Lee-Fay Low
A/Prof Robyn Woodward Kron
Dr Jim Hlavac Dr Joanne Enticott
Dr Josefine Antoniades
Dr Andrew Gilbert
Dr Xiaoping Lin
Dr Jenni White
Mr Kerry Hwang

Sponsors:

NHMRC Partnership Projects 2020
National Accreditation Authority for Translators and Interpreters (NAATI)
Australian Institute of Interpreters and Translators (AUSIT)
Dementia Australia
Migrant and Refugee Health Partnership
All Graduates Interpreting and Translation Services
Translating and Interpreting Service (TIS National)
Healthcare Interpreter Service (NSW) though the Sydney Local Health District

Confidential

This document is confidential and the property of National Ageing Research Institute. No part of it may be transmitted, reproduced, published, or used without prior written authorization from the institution.

Statement of Compliance

This document is a protocol for a research project. This study will be conducted in compliance with all stipulation of this protocol, the conditions of the ethics committee approval, the NHMRC National Statement on ethical Conduct in Human Research (2007) and the Note for Guidance on Good Clinical Practice (CPMP/ICH-135/95).

This is the original ethics-approved protocol for the entire MINDSET study. Study 2 is the protocol specifically for the MINDSET trial.

Table of Contents

Study synopsis 3
Glossary of abbreviations and terms 4
Study sites 5
1 Introduction and background 6
1.1 Lay summary 6
1.2 Introduction 7
1.3 Background information 7
2 Study objectives 9
2.1 Project aims 9
2.2 Hypotheses 10
2.3 Theoretical framework 10
3 Project governance and steering group 11
4 Study 1 – Co-design of the training 12
4.1 Review of literature and existing training 12
4.2 Qualitative interviews ....**Error! Bookmark not defined.**
4.2.1 Participants and recruitment ....**Error! Bookmark not defined.**
4.2.2 Method 12
4.3 Co-design workshops 12
4.3.1 The World Café methodology 13
4.3.2 Workshop participants and recruitment 13
4.3.3 Workshop schedule 14
4.4 Development of training and education materials......................................15
4.5 User testing..15
4.5.1 Participants...15
4.5.2 Method 15
5 Study 2 – Randomized control trial of training 16
5.1 Participants 16
5.2 Randomisation..16
5.3 Intervention 16
5.4 Outcome measures 17
5.5 Sample size justification...18

5.6 Statistical analysis 18
6 Study 3 – Implementation of training 19
6.1 Promotion of the training to interpreters 19
6.2 Monitoring 19
6.3 Evaluation 19
6.3.1 Participant eligibility 20
7 Participant Safety and Withdrawal 21
7.1 Consent 21
7.2 Risk management and safety 22
7.3 Handling of withdrawals 23
7.3.1 People with dementia 23
7.3.2 Family carers 23
7.3.3 Interpreters 24
7.3.4 Clinicians 24
7.4 Replacements 24
7.4.1 Study 1 24
7.4.2 Study 2 24
7.4.3 Study 3 24
8 Data Security and Handling 25
8.1 Details of where records will be kept and how long will they be stored 25
8.2 Confidentiality and security 25
8.2.1 Study 1 25
8.2.2 Study 2 25
8.2.3 Study 3 26
8.3 Ancillary data 26
8.4 Public availability of data 26
9 References 27

Study synopsis

**Title:**	Improving interpreting for dementia assessments: The MINDSET Study
**Short title:**	MINDSET
**Study centres:**	National Ageing Research Institute, universities, and interpreting services
**Study aim:**	To develop, user-test, evaluate, and implement online dementia assessment training for language interpreters.
**Primary objectives:**	1. To improve the quality of interpreter communication in cognitive assessment for dementia.<br>2. To implement the training in Victoria, assessing for scalability and services' satisfaction.
**Design:**	Study 1: Co-design the online training modules with interpreters, clinicians and carers of Culturally and Linguistically Diverse (CALD) people with dementia (Aim 1).<br>Study 2: Undertake a national randomised control trial (RCT) with 150 interpreters to assess in the impact of online training on the quality of interpreter communication in comparison to wait-list control (Objective 1).<br>Study 3: Implement the training in Victoria focusing on scalability, and assess services' satisfaction with the training via qualitative interviews with interpreters, managers, and clinicians to (Objective2).
**Outcomes:**	Study outcomes realise a key priority in the NHMRC's National Institute of Dementia Research CALD Action Plan, which is to, "Inform effective ways to train frontline ... staff on how culture influences dementia." With a >200% projected growth in the proportion of older CALD Australians by 2056, there will be a rise in the number of non-English speaking people living with dementia. The national benefit of this study is that it will enable interpreters to be prepared and confident in performing their role impartially, effectively, and accurately. The training would ultimately sit on the National Accreditation Authority for Translators & Interpreters (NAATI) website and provide opportunity for every interpreter working with older CALD Australians to access training in interpreter mediated cognitive assessment for dementia.
**Safety considerations:**	Informed consent required from all participants.

Glossary of abbreviations and terms

Abbreviation/term	Description
Interpreter	Person working professionally in the capacity of providing language interpreting services
RCT	Randomised control trial
NARI	National Ageing Research Institute
NHMRC	National Health and Medical Research Council
NAATI	National Accreditation Authority for Translators and Interpreters
AUSIT	Australian Institute of Interpreters & Translators
CALD	Culturally and Linguistically Diverse
CPD	Continuing Professional Development
CDAMS	Cognitive Dementia & Memory Service
ACAS	Aged care assessment service
PICF	Participant Information and Consent Form
HREC	Human Research Ethics Committee
CI	Chief investigator

Study sites

Site	Address	Contact/s
University of Western Australia	[details removed]	Prof Bianca Brijnath<br>[details removed]
National Ageing Research Institute (NARI)	[details removed]	Dr Andrew Gilbert<br>[details removed]<br>A/Prof Bianca Brijnath<br>[details removed]
National Accreditation Authority for Translators and Interpreters (NAATI)		Mark Painting<br>[details removed]
RMIT University		Dr Erika Garcia Gonzalez<br>[details removed]

1 Introduction and background

1.1 *Lay summary*

This project will develop, test and implement a training course (“MINDSET”) for language interpreters who work with people living with dementia, or interpret for cognitive testing. Aged care assessment guidelines require that an interpreter be present when older people from non-English backgrounds undertake a cognitive test and interpreting for these assessments makes up a significant portion of many interpreters’ work schedule. However, interpreters can face challenges with the terminology of cognitive tests as well as working with older clients whose communication skills are affected by dementia or cognitive impairment. We will therefore develop a National Accreditation Authority for Translators and Interpreters (NAATI) accredited training course aimed at improving interpreters’ competence and confidence in this. The project consists of three studies, over three years.

During the first study, we will co-design the material for the training course with relevant stakeholders and then conduct user-testing of the finished course with interpreters. We will invite ~25 participants, including interpreters, clinicians, people living with dementia, and family carers, to participate in two workshops, over two days. The research team will design the course based on input from these workshops and based on what we know from other research. Once developed, the course will consist of 3-5 modules, take interpreters 3-4 hours to complete, and be accessible online through the NAATI website. We will also design course assessments, which will involve both quizzes and simulated interactions of cognitive assessments. Ten interpreters will take the course and assessments and provide feedback to the researchers for further refinement.

The second study will be a RCT (randomized control trial) to measure whether the training course improves interpreters’ performance when interpreting for cognitive assessments. The trial will involve two groups of 75 interpreters (150 in total), recruited through our interpreting industry partners and then randomly assigned to each group. The intervention group will undertake the training and the control group will not receive the training until after the trial is over. Interpreters from both groups’ will be assessed at three intervals, at the beginning of the trial (baseline), after 3-months (post-intervention) and after 6-months (follow up). The assessment will involve a 40-minute simulation of a cognitive assessment and questionnaires about medical terminology, ethics, medical interpreting, and cross-cultural communication. We will combine each interpreters’ assessment results at each interval into a composite score. We will then statistically analyse whether there is an improvement in performance after doing the training, as well as differences between the intervention and control groups.

The third study will be an implementation of the training course, which will make it available to all Victorian interpreters. Lessons from this study will guide our national implementation strategy. We will monitor training uptake with a target of 70% of eligible Victorian interpreters completing the course. After 50% of our target (i.e. 35% of eligible Victorian interpreters have done the training) is complete, we will conduct ~20-30 interviews with interpreters and interpreting agency managers to discuss training uptake, barriers and facilitators to the training, and the effect on interpreters’ work practices. We will also interview ~20 clinicians who frequently work with people with dementia from CALD

backgrounds to understand how the interpreters' training has influenced their work practices and their experiences with interpreters.

1.2 *Introduction*

This project will develop the first dementia assessment training for interpreters in Australia. The training aims to improve interpreters' competence and confidence in working with older people undertaking cognitive assessments, and thereby facilitate better accuracy of assessments. By extension, this aims to facilitate timely diagnosis of dementia among Australia's ageing culturally and linguistically diverse (CALD) populations and improve access to dementia-related services for older people from CALD backgrounds.

We will co-design with interpreters, clinicians, people with dementia and their carers online training modules for interpreters. These training modules will: describe dementia and its impact on cognitive and linguistic ability; explain the tools used to assess and diagnose dementia; and engage interpreters with effective interpreting strategies for cognitive assessments. The impact of the training on interpreter's quality of communication will be evaluated using a randomised controlled trial.

The key objectives of this project are to:

- Improve the quality of interpreter communication in cognitive assessment for dementia.
- Implement the training in Victoria to plan for national scale-up.

To achieve the objectives of this project, we will conduct three studies over three years, in which we will:

1) Co-design with stakeholders a series of training modules for interpreters on interpreting for dementia.
2) Assess the effectiveness of the training modules through a randomized control trial.
3) Monitor and evaluate the implementation of the training modules across the Victorian interpreting sector.

Once this project is complete, the training modules will be made available to every accredited interpreter in Australia. The training modules will be maintained on our industry partner, the NAATI website and will be integrated in the mandatory Continuing Professional Development (CPD) for interpreters. Interpreters will be able to access and complete the training at any time.

1.3 *Background information*

Australia has an ageing migrant population. With the number of CALD Australians diagnosed with dementia increasing each year, there is growing demand for language interpreters for cognitive assessments and dementia-related health consultations. In order to ensure accuracy and impartiality, NHMRC (National Health and Medical Research Council) Dementia Clinical Guidelines specify that a professional interpreter should be used during cognitive assessments with an older person whose first language is not English [1]. Use of professional interpreters rather than informal interpreters is known to facilitate greater

accuracy, better communication, higher satisfaction from clients and clinicians, and can mitigate concerns that interpretation by non-professionals may involve conflicts of interest [2, 3].

However, even professional interpreters can influence assessments, although the reasons are poorly understood [4-6]. Interpreting for cognitive assessments requires a high degree of accuracy in communicating specific questions, instructions, and/or medical terminology to clients. Communication with cognitively impaired clients can also pose specific challenges, as participants often rely on extra-verbal bodily gestures, facial expressions, and timing to maintain a focused interaction [7-9]. Furthermore, some CALD patients have low health literacy and require reassurance and support in understanding the purpose and process of undertaking a cognitive assessment [5, 6]. Professional interpreters face uncertainties in how to negotiate these demands [8], potentially leading to communication that compromises assessment accuracy and/or creates misunderstanding between patients and clinicians [3, 9]. A lack of specific training in dementia means interpreters may not have the skills for communicating with cognitively impaired patients, knowledge of cognitive assessments, and an understanding of their role in assessments [4, 6, 8, 9].

Prior work by members of this project’s research team has identified a demand for specialized training in dementia for interpreters. We have found that difficulties in translation and interpretation contribute to delayed diagnoses of dementia and delayed uptake of health and care services [10]. Cognitive Dementia and Memory Services (CDAMS) and Aged Care Assessment Services (ACAS) rely on hospital- and community-based interpreters when assessing non-English proficient clients [11]. However, interpreting services in Australia are subject to supply and demand imbalances, inconsistent quality of services, and lack of availability in regional areas [12, 13]. This means that while some CDAMS and ACAS can access hospital interpreters in some languages, many assessment services (especially in regional and rural areas) must rely on community interpreters to interpret cognitive assessments for dementia.

In Australia, there currently exists no specialized training for community interpreters working with clients living with dementia or undergoing cognitive assessment. A community-based interpreter workforce familiar with dementia and dementia assessments will be required to meet the growing demand for assessments owing to ageing migrant population. For many community interpreters, dementia-related medical appointments and aged care assessments are a regular source of work. Specialized interpreter training in dementia and cognitive assessments will, we argue, ensure these interpreters are confident and prepared when servicing this clientele. The training will also support the overall accuracy and validity of cognitive testing for older people from CALD backgrounds.

2 Study objectives

2.1 *Project aims*

The MINDSET project's aims are:

1. To improve the quality of interpreter communication in cognitive assessment for dementia.
2. To implement the training in Victoria, assessing for scalability and services' satisfaction.

We will achieve our project aims by conducting three studies (Fig.1):

Study 1

Co-design the online training modules with interpreters, clinicians, and carers of CALD people with dementia via co-design workshops (Aim 1).

Study 2

Undertake a national randomised control trial (RCT) with 150 interpreters to assess in the impact of online training on the quality of interpreter communication in comparison to wait-list control (Aim 1).

Study 3

Implement the training in Victoria focusing on scalability and assess services' satisfaction with the training via qualitative interviews with interpreters, managers, and clinicians to (Aim 2).

Fig.1: MINDSET project

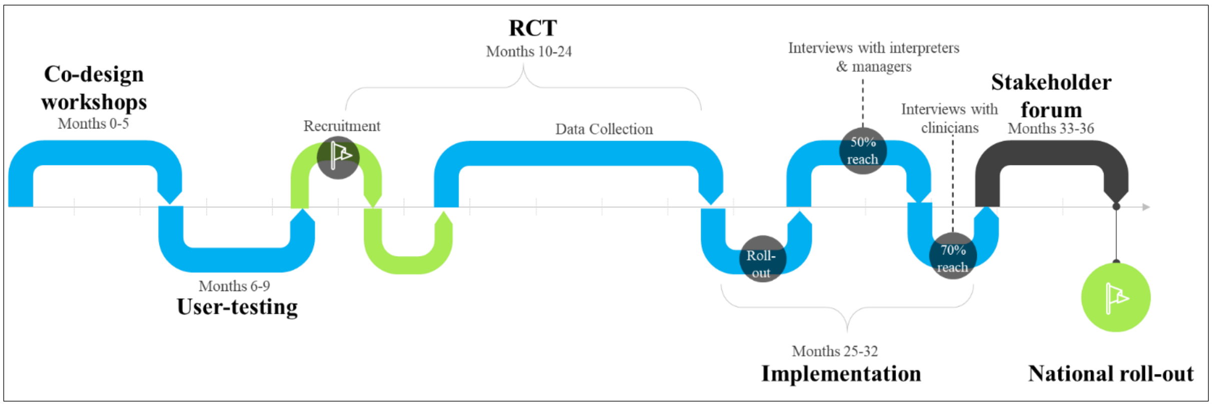

2.2 *Hypotheses*

Our hypotheses are that:

Study 1

1) Interpreters, clinicians, and carers of CALD people with dementia will, together with the research team and industry partners, successfully co-design training modules that improve the quality of interpreted dementia assessments.

Study 2

2) Interpreters trained in the MINDSET program will show an improvement in the quality of interpreted communication for dementia assessments at 3 months and 6 months following the intervention, when compared to their baseline scores.
3) Interpreters trained in the MINDSET program will show a greater improvement in the quality of interpreted communication for dementia assessments at 3 months and 6 months following the intervention, when compared to the control group's scores.

Study 3

4) The MINDSET program will be found to be feasible, acceptable, and effective by interpreters, their managers, and clinicians who frequently work with CALD clients in dementia related services.

2.3 *Theoretical framework*

We apply the RE-AIM [14] theoretical framework to co-design an intervention that creates the implementation outcomes necessary for achieving a sustainable and scalable intervention [15] (Table 1). This framework has been used in over 430 studies to develop public health initiatives and considers the following elements: reach, effectiveness, adoption, implementation, and maintenance [15].

Table 1. Application of the RE-AIM framework in MINDSET

Theory	Method	Activities
Reach	Qualitative interviews, Co-design workshop, RCT, Implementation study	We will assess how co-design methods strengthened the reach and uptake of the training and assess the extent to which interpreters remain engaged via the RCT and implementation study.
Effectiveness	RCT	We will assess the extent to which the training intervention effected interpreter communication quality.
Adoption	Co-design, Implementation study	We will work with interpreters and clinicians to identify during the co-design and implementation how the training will be applied in practice.
Implementation	RCT, Implementation study	We will identify factors that are likely to influence scale-up of the training and use the lessons learned to advise our partners for national scale-up.
Maintenance	Knowledge transfer.	We will give the training package to NAATI and promote it among our industry partners to maintain the training.

3 Project governance and steering group

A project Steering Group has been formed, consisting of our industry partners, Chief Investigators (CIs), two CALD consumers, two interpreters, and the Project Manager. The Steering Group will meet twice a year. This group will provide oversight of the research, offer guidance about the appropriateness and applicability of the intervention, and ensure that the intervention aligns with concurrent and planned policy and regulatory initiatives. The Steering Group will also meet for the co-design workshops and for a Stakeholder Forum towards the end of the project. A project Executive Team will include the CIs, AIs, and Project Manager. The Executive Team will be responsible for implementation of the project and all related activities. The Project Executive Team will meet every second month. The Implementation Team, consisting of CI Brijnath, the Project Manager, and the NAATI assessors (Study 2/Year 2) will meet monthly.

The project is a partnership between the research team, key representatives from the interpreting industry, Dementia Australia, and the Migrant and Refugee Health Partnership. It has funding from several sources, including our interpreting industry partners and through a grant from the NHMRC Partnership Projects 2020 (APP2005759). Industry funding partners will be involved in the project in the Steering Group in an advisory capacity and will support recruitment efforts and dissemination of the final product. However, funding partners will be not involved in the collection or analysis of data, nor in the reporting of project outcomes, except for data gathered by NAATI during the monitoring phase of the implementation study (see section 6.2).

4 Study 1 – Co-design of the training

Study 1 will use information gathered from multiple sources and apply a co-design approach to develop an evidence-based, co-developed and user-tested training package. Study 1 will therefore involve the following stages:

1.1) Review of literature and existing training
1.2) Co-design workshops with stakeholders
1.3) Development of training and education materials
1.4) User-testing of training

4.1 *Review of literature and existing training*

A comprehensive review of any literature on language interpreting for people with dementia, during dementia-related consultations, or of cognitive assessments will be conducted using a scoping study methodology [16]. We will also conduct an environmental scan to identify any existing training materials or interventions designed to increase interpreters' competencies and knowledge when working with clients with dementia or interpreting for cognitive assessments. This review will ensure the development of training materials is informed by the latest evidence, and it will support us with mapping out how training materials will be targeted toward achieving the project aims.

4.1.1 Method

Once consent has been obtained, interviews will take place either in-person, over Microsoft Teams, or over telephone and will be audio-recorded. The interviews will be short and flexible - up to 30 minutes in duration – and will take into the needs of the person with dementia and the availability of any support they require. People with dementia may choose to complete the interview with a family carer or support person present. However, they will not be required to have a carer or support person present if they do not wish to have one. Interviews will consist of questions about the participants' experiences with interpreters, what interpreters did well to support their needs, what interpreters could improve upon, and any other things that interpreters should know when interpreting for people living with dementia.

We recognize there is a potential conflict of interest if interpreters are used for these interviews. For this reason, participants will be interviewed by bilingual NARI researchers, who have previous qualitative interviewing experience from the *Moving Pictures* project (Curtin University HRE2017-0758). Participants will be given the choice of whether they speak in English or their other primary language during interviews.

4.2 *Co-design workshops*

This stage recognises the importance of working with interpreters, clinicians, and family carers to better understand their mutual needs and expectations, and thereby develop training that allows interpreters to perform optimally in their role. Using a co-design technique ensures that the MINDSET program will genuinely meet the needs of older clients, and the result will be a program more relevant to all stakeholder due to the fact that they have been involved in the design and evaluation of it.

Co-design emphasises the importance of lived experience, collaboration, and participation of all stakeholders in the research [17]. Interpreters, clinicians, and carers will collaborate to identify and address the challenges and issues from multiple perspectives, and design solutions to enhance desirability, acceptability and usability of the training package [17].

Workshops will be conducted online (via Teams) to minimize the risk of the project being delayed by any COVID-19-related restrictions. Online workshops also overcome the need for participants to travel to a central location, which enables better access for some participants.

4.2.1 The World Café methodology

The workshops will be conducted online using the World Café methodology, which is a type of focus group that enables participants to be part of evolving rounds of dialogue with three or more people while simultaneously being a part of an interconnected conversation with the other groups at the workshop [18]. The World Café methodology regards all participants as experts of their own lived experience and life-worlds, and the focus is on encouraging the sharing of diverse perspectives rather than reaching consensus. The method is designed to facilitate a conversational greenhouse in which there is a rapid propagation of actionable knowledge and collective solutions [18].

The World Café methodology has been demonstrated to promote high quality consumer engagement and meaningful collaboration between stakeholders [19]. However, in order to mitigate the possibility that power imbalances between different participant groups (e.g. interpreters, clinicians, and people with dementia) means some views less heard than others, we will periodically use break out rooms to separate groups and allow space for them to reflect on their specific knowledge and experiences [20]. The research team will also encourage equal participation throughout the workshops, and actively manage group dynamics and conflict resolution.

We will utilize the Microsoft Teams inbuilt recording function to record workshops, provided that participants' voluntarily consent to being recorded. Research team members will also be present throughout the workshops, taking field notes of the conversations, and recording the outcomes of activities. Following the workshops, all records will be analysed by members of the research team for the purpose of designing the training modules. Workshop recordings may be transcribed, depending on project resources, and requirements. All notes, recordings and transcriptions will be subject to the project's data security and handling policy.

Participants will connect to the workshops via Microsoft Teams. We will assign participants to break out rooms, first placing participants of the same stakeholder group (e.g. interpreters, clinicians, and carers) together, then in rooms with a mix of stakeholders from a range of backgrounds and perspectives. CIs Brijnath, Low, and Antoniades have successfully used these methods in their current research [21-23].

4.2.2 Workshop participants and recruitment

Alongside the research team and partners, up to 25 stakeholders will participate in the co-design workshops. Participant groups will comprise:

- 6-8 interpreters
- 5-7 clinicians (e.g. geriatricians, neuropsychologists, old age psychiatrists).
- 5 - 7 CALD family carers

<u>Interpreters</u> will be recruited for workshops through our interpreter industry partners, NAATI and AUSIT, who will use their internal mailing lists to issue calls for expressions of interest with contact details of the research team. To be eligible, interpreters must be NAATI credentialed with provisional certification or professional certification and 6+ months practicing experience. Interpreters will be remunerated by our industry partners according to a standard hourly interpreting rate for time spent participating in workshops.

CALD carers will be recruited for workshops by the NARI research team. NARI has contact with potential candidates who were previously recruited as participants in the *Moving Pictures* project, in accordance with that project's ethics protocol (Curtin University HRE2017-0758). NARI researchers will contact some of these candidates directly and issue a call for expressions of interest through the regular *Moving Pictures* newsletter. Any recruits will be required to review and complete a MINDSET written consent form. Each participating carer will be offered one $100 gift card as an expression of gratitude for the time they spend in both workshops.

Clinicians will be recruited through the Australian Dementia Network – a professional network of clinicians and researchers. CI Low is a member of the network. A call for expressions of interest, with contact details of the research team, will be issued through the network's internal mailing list. Clinicians will be offered $500, $250 per a workshop, for their participation. This is what we estimate it will cost to remunerate qualified medical professionals (e.g. general practitioners, specialists) for their time, including any backfill and other expenses they or their organizations incur from their participation in our workshops. If they are only able to attend one workshop, by arrangement with the research team, they can be compensated $250.

4.2.3 Workshop schedule

The co-design will comprise two half-day workshops, approximately 2 weeks apart. The workshop agendas will be scheduled as follows:

4.2.3.1 Workshop 1

Participants will be introduced to dementia. Then Participants will convene in break-out rooms and asked to identify the most challenging aspects of interpreter-mediated dementia assessment, and exemplars (existing or possible) of interpreter training initiatives that address these challenges. Participants will be allocated to break-out rooms according to their role (e.g. interpreters, clinicians, family carers).

Then participants will reconvene and they will be presented with the five interpreting domains critical to quality communication, as identified by international research [24, 25]:

a) Phraseology: identifying frequently used phrases in cognitive assessment and medical encounters,
b) Terminology: specific terminology,
c) Code of ethics: knowledge and application of the relevant interpreter code of ethics,
d) Contextual knowledge: medical interpreting in various situations (reception, cognitive assessment, medication administration guidance, and informed consent);
e) cross-cultural communication.

Participants will be informed that the training will be designed around these five aims and they will be asked to comment and endorse them.

Then Participants will convene in break-out rooms in mixed groups to elaborate on preferred types of pedagogical learning to enhance uptake of the training.

4.2.3.2 Workshop 2

We will reconvene participants from Workshop 1 and present them with the training program (addressing the interpreting domains), the training structure (addressing preferred teaching format), and the simulation scenario (through which the impact of the training will be assessed). After each component of the training is presented, Participants will be divided into

mixed break-out rooms, and asked to comment on the proposed training content, identify the value proposition of the training, and develop the key messages most effective at explaining the training. Detailed recordings and notes from the workshops will be collected by the research team, compiled into one central list, and used to refine the training modules.

4.3 *Development of training and education materials*

The research team will use ideas and feedback generated during the co-design workshops to develop the training package. Training will comprise 3-5 modules of 3-4 hours duration, incorporating multimedia and text-based learning as well as didactic and interactive learning (e.g. lectures, quizzes etc.). CIs Brijnath, LoGiudice, Low, and Antoniades have experience in developing and implementing dementia training, CIs Gonzalez and Hlavac in interpreter training, and CI Woodward-Kron in simulation training for health professionals. The training content and the simulation script, which will be used to assess the training, will be developed by the research team. It will then be operationalised for an online learning environment by web architects (Elbury e-learning), designers (Ooi), and filmmakers (Fire Films) with whom we have ongoing links through our related CALD-dementia studies.

4.4 *User testing*

Once the online training and simulation assessments have been developed, they will be user-tested by 10 interpreters as part of process evaluation.

4.4.1 Participants

10 interpreters will be recruited for user testing through our interpreter industry partners, TIS National, who will use their internal mailing lists to issue calls for expressions of interest with contact details of the research team. To be eligible, interpreters must be NAATI credentialed with provisional certification or professional certification and 6+ months practicing experience. Interpreters will be remunerated by our industry partners according to a standard hourly interpreting rate for time spent participating in user-testing.

4.4.2 Method

Interpreters will undertake the training including the simulation assessment. Using the thinking-aloud method [26] and a 60-minute exit survey, we will assess how long the training takes to complete, it's acceptability, including most/least relevant and helpful aspects, the main barriers and facilitators of using the online platform, and elicit any recommended improvements. These assessments will be undertaken by researchers at NARI and RMIT.

A bespoke survey questionnaire will be developed for the assessments based on the training modules' content and guided by the Unified Theory of Acceptance and Use of Technology [27]. The survey will assess direct determinants of intention. These include: performance expectancy (the degree to which using the training will provide benefits in performing an interpreting role), effort expectancy (the degree of ease associated with the use of the training), and social influence (the degree to which an interpreter perceives that others important to them believe they should complete this training).

This process evaluation in combination with the research team's observations and partners' industry experiences will be collated to finalise the training modules for trial evaluation, implementation, and roll-out.

5 Study 2 – Randomized control trial of training

Study 2 will be a randomized control trial (RCT), conducted to evaluate whether the training package developed during Study 1 fulfils our primary project aim of improving the quality of interpreter communication in a cognitive assessment for dementia. The trial will be a 6-month wait-list RCT with a parallel design, conducted with a target of 150 interpreters in equal allocation (75 in the intervention group and 75 in the control group).

Outcomes will be assessed at baseline (t_0), post-intervention (3m after baseline; t_1) and follow-up (6m after baseline; t_2). The baseline covariates will include characteristics of interpreters (age; sex; ethnicity; location; educational and economic attainments; and NAATI credential status).

5.1 *Participants*

150 interpreters will be recruited for the trial through our interpreter industry partners, NAATI and AUSIT, who will use their internal mailing lists to issue calls for expressions of interest with contact details of the research team. To be eligible, interpreters must be:

a) NAATI credentialed with provisional certification or professional certification;
b) Have 6+ months practicing experience;
c) Have access to an internet connection;
d) Interpret for Greek, Italian, Mandarin, Cantonese, Arabic or Vietnamese languages; and
e) Not participated in study 1 (in either the workshops or the user-testing).

5.2 *Randomisation*

Interpreters will be randomised into:

a) an <u>intervention group</u> (*n*=75*)* who will use the training package for 6m immediately following the randomisation, or
b) a <u>wait list control group</u> (*n*=75) who will receive access to the training package six months after randomisation.

This approach was chosen because it involves no deception of participating interpreters, and because both groups of interpreters will eventually have access to the training.

After enrolment in the trial, and before randomisation, participants will complete the simulated battery to measure the primary outcome of quality of interpreted communication. The test outcome will not be provided to the person until an external statistician has done the randomisation procedure (randomisation by minimisation). A researcher at National Ageing Research Institute will provide the test outcome and randomisation outcome (test/control) to the person approximately one week after doing the simulated tests. This ensures that randomisation outcome is independent of baseline scores. In this pragmatic trial, it is not possible to blind participants to group allocation when collecting the post-intervention scores. Hence, this trial is a single-blinded study where outcome assessors will be blinded to allocation.

5.3 *Intervention*

Interpreters in the intervention group will have access to the intervention for 3 months from immediately after randomisation. Adherence is defined as a participant completing 70% of the training. The wait list control group will receive access to the training after completing the 6-

month assessment (t_2). All those who complete the training package will receive Continuing Professional Development points that count towards NAATI interpreter certification.

5.4 *Outcome measures*

Our primary outcome is the quality of interpreted communication measured via a battery composite *z score* evaluating six interpreting domains [24, 25] crucial to communication quality (Table 2). These measures will be collected using questionnaires, scenario response testing and video footage of a virtual simulated patient consultation. Simulation pedagogy is a core element of clinical training in health professional education [28] as it provides a structured and safe environment allowing performance review and development. Simulations have been used to evaluate practice in interpreter studies [29].

Table 2: Quality of interpreted communication measured via a battery composite *z* score

**Domains**	**Measures**	**Collected via**
1. Pre-interactional briefing with clinician; explanation of role to clinician and limited English proficiency client	Simulations to be developed based on input from co-design, literature review, and *Mental Health Interpreting Guidelines for Interpreters* [30]	Video footage rated by an experienced interpreter
2. Accurate and complete bi-directional meaning transfer inter-lingually; interactional management skills; rhetorical skills; linguistic competency and accuracy.	Simulations to be developed based on input from co-design, literature review, NAATI Certified Interpreter Test Assessment Rubrics [31] and *Mental Health Interpreting Guidelines for Interpreters* [30]	Video footage rated by an experienced interpreter.
3. Knowledge of medical terminology	Dementia-Knowledge Assessment Scale[28]	Questionnaire
4. Knowledge of dementia diagnosis scales and dementia-related services.	Simulations and questions to be developed based on co-design and literature review.	Questionnaire and video footage
5. Working ethically with clinicians and people living with dementia	Simulations and scenarios to be developed based on input from co-design, literature review, AUSIT code of ethics[29] and *Mental Health Interpreting Guidelines for Interpreters* [30]	Scenario-response test and video footage
6. Demonstration of intercultural competency	Simulations, scenarios and questions to be developed based on input from co-design and NAATI Intercultural Competency Text Rubrics [32]	Questionnaire, scenario response test, and video footage

As the content of the video simulations and questionnaires depend upon input gathered through interviews and workshops during study 1, the measures will be developed after study 1 is complete. CIs Gonzalez and Hlavac, who both have expertise in the training and assessment of interpreters, will lead development of measures. They will ensure measures adhere to NAATI assessment standards and relevant interpreter ethical guidelines. Once the

measures have been fully developed, they will be submitted to the HREC for approval as an amendment prior to any study 2 data being gathered.

In our study, the simulation of ~40 minutes will mimic a typical cognitive assessment of a CALD patient at CDAMS/ACAS (Box 1). The patient and clinician's dialogue will be uniform, pre-scripted, and filmed in the top six languages spoken by older CALD Australians: Greek, Italian, Mandarin, Cantonese, Vietnamese, and Arabic. The interpreter's dialogue is not scripted, and they will be required to interpret and facilitate the assessment as they would in a standard consultation. Interpreters will be asked to click a video record button to record their online simulated consultation (similar to how Zoom meetings are recorded). These simulations will be automatically saved and uploaded, ready for assessment. After completing the simulation, interpreters will complete the questionnaires. Video footage and the questionnaires will be assessed by blinded assessors who are NAATI examiners, seasoned in applying this assessment model regularly. To ensure rigour, CI Hlavac will train the assessors about the assessment items and each assessor will have their first four assessments double assessed to check for inter-rater reliability. In scenarios where components of the simulation are missing or incomplete, assessors will only award marks to the overall score for sections of the cognitive assessment that were interpreted. Failure to interpret the introductory or concluding component of the assessment will not affect the score.

Box 1: A standard CDAMS/ACAS

- Case briefing between the clinician and interpreter (5mins)
- Introduction to the patient (5mins)
- Interpretation of the RUDAS (15mins)
- Ask about medical issues, mood etc. (5mins)
- Next steps (5mins)

5.5 *Sample size justification*

The primary outcome is a score calculated using the method described above and based on that done by Ono et al [24]. A sample size of 120 (60 test and 60 control) participants (interpreters) is sufficient to detect with 80% power and alpha of 0.05, a difference in control and test group means of 1.3 with standard deviation of 2.5 (i.e. means of 2.91 control and 4.21 test in Ono et al[24]). The calculations are the customary ones based on normal distributions[33]. The target sample size is set at 150 interpreters (75 test and 75 control) to include oversampling for participant withdrawals, and because we see no foreseeable adverse effects for either group resulting from participation in the trial.

5.6 *Statistical analysis*

To evaluate our primary outcome, we will use mixed effects generalised linear regression. Random effects will account for repeated measures from participating interpreters. Time-point (baseline, 3m and 6m) will be a categorical variable and specified as fixed. Other independent variables will be specified as fixed (intervention/control, age; sex; ethnicity; location; educational and economic attainments; and NAATI credential status).

6 Study 3 – Implementation of training

In Study 3, we will follow World Health Organization guidance for scaling up health service innovations [34] to implement the training among eligible Victorian interpreters. By doing so, we will monitor, evaluate, and adapt the training to real-world circumstance, and then use the lessons learned to guide our national and inter-state partners for Australia-wide implementation.

Following study 2, the training will be rolled out to all NAATI certified interpreters across Victoria. We will conduct two rounds of qualitative semi-structured interviews: first with interpreters and managers while implementation is underway and second with clinicians once implementation is complete.

6.1 *Promotion of the training to interpreters*

There are currently 1,613 NAATI interpreters in the nominated languages based in Victoria. To reach them and encourage them to take up the training, we will use multiple channels to tell a compelling story and build coalitions and networks to promote the training [34]. Eligible interpreters will be identified and formally invited by NAATI to complete the MINDSET training. Promotional messages about the MINDSET training will also be circulated by AUSIT, All Graduates, and Dementia Australia. Interpreter testimonies will be used to build motivation and emails will be sent to the CEOs/relevant heads of interpreter agencies (e.g. Translating and Interpreting Services (TIS National), Language Loop, Polaron, On Call), with whom we have established relationships, to inform them about the free training, and ask them to encourage their eligible interpreters to participate.

These strategies will be repeated at different points during the implementation period. All interpreters who complete the training will receive CPD points.

6.2 *Monitoring*

We will monitor implementation in Victoria through data collected as part of NAATI's online training platform, where the training modules are hosted. The following data will be collected:

a) Baseline covariates of interpreters taking up the training (age, sex, ethnicity, location, educational and economic attainments, and NAATI credential status)

b) Total uptake (number of completions),

c) Rate of uptake (completions/per week),

d) Geographic coverage (via postcode).

The training will be considered implemented when 70% of eligible Victorian interpreters (n=1,129) have completed the training. The monitoring will help us identify any barriers to implementation and to assess whether economies of scale are being reached (i.e. 70% coverage across metro and regional areas).

6.3 *Evaluation*

To understand the process of implementation, once we reach 50% of our target (i.e. 35% coverage) we will begin telephone/video interviewing 20-30 interpreters and interpreter agency managers to understand: training uptake, potential facilitators/barriers and solutions; and whether the essential features of the training are being adhered to in practice (and if not, what remedial action can or should be taken). This will allow us to make necessary adjustments.

Once we reach 70% of our target (i.e. 50% coverage), ~20 clinicians from high CALD caseload CDAMS will be interviewed to understand if the training improved interpreter communication, impacted practice, and clinician satisfaction.

6.3.1 Participant eligibility

Interpreters

Eligible interpreters who have completed the training will be identified by NAATI and invited to participate in interviews. To be eligible for interviews, interpreters must:

a) Be NAATI credentialed with provisional certification or professional certification;
b) Have 6+ months practicing experience;
c) Have access to an internet connection;
d) Interpret for Greek, Italian, Mandarin, Cantonese, Arabic or Vietnamese languages;
e) Reside in Victoria; and
f) Have not participated in studies 1 or 2.

Interpreters will be remunerated by our industry partners according to a standard hourly interpreting rate for time spent participating in interviews.

Managers

The research team will contact eligible managers from key interpreting agencies to take part in interviews. To be eligible managers must be working in a leadership role at an interpreting agency in Victoria with oversight of interpreter CPD.

Clinicians

There are 21 Cognitive Dementia & Memory Service (CDAMS) clinics in Victoria (11 Melbourne/10 regional). We will obtain names and contact details of high CALD caseload CDAMS from All Graduates. High caseload clinics will be sent an email describing the study and inviting their clinicians to participate in an interview. Non-responders will get a reminder letter and phone call.

7 Participant Safety and Withdrawal

7.1 *Consent*

The research staff who contact people living with dementia during study 1 will be trained to recognise signs that the person does not fully understand the implications of participating in research. For example, the potential participant may think they are receiving a service from NARI or they may not be able to sufficiently orient themselves to time and place to be able to schedule an appointment for the interview. When conducting qualitative interviews, the researcher will again assess the potential participant's capacity to give consent.

Capacity to provide consent will be determined by whether the potential participant:

- Understands the nature of the research and their participation in the research;
- Appreciates the consequence of their participation;
- Shows the ability to consider alternatives including the option to not participate;
- Shows the ability to make a reasoned choice.

Written and verbal techniques will be used to communicate the details of the research, and the potential participant will be asked to explain the details back to the researcher. This approach is recommended by the Dementia Collaborative Research Centres as cognitive decline can be specific to one or more cognitive processes and a general cognitive screen may not be the best way to judge a person's capacity to give consent to participate in research.

Researchers will follow the ‘Decision Tree for Respecting Dissent and Seeking Assent for Dementia Research’ [35] (See Figure 2) throughout recruitment and interviews with participants living with dementia. If the researcher thinks that capacity is impaired and informed consent is not valid, then the potential participant will be politely dissuaded from participating in the study and offered instead to be put on the NARI mailing list to receive a quarterly newsletter. This approach is considered less invasive than conducting a formal cognitive test with someone who may not have capacity to consent to the test or involving the potential participant’s medical practitioner.

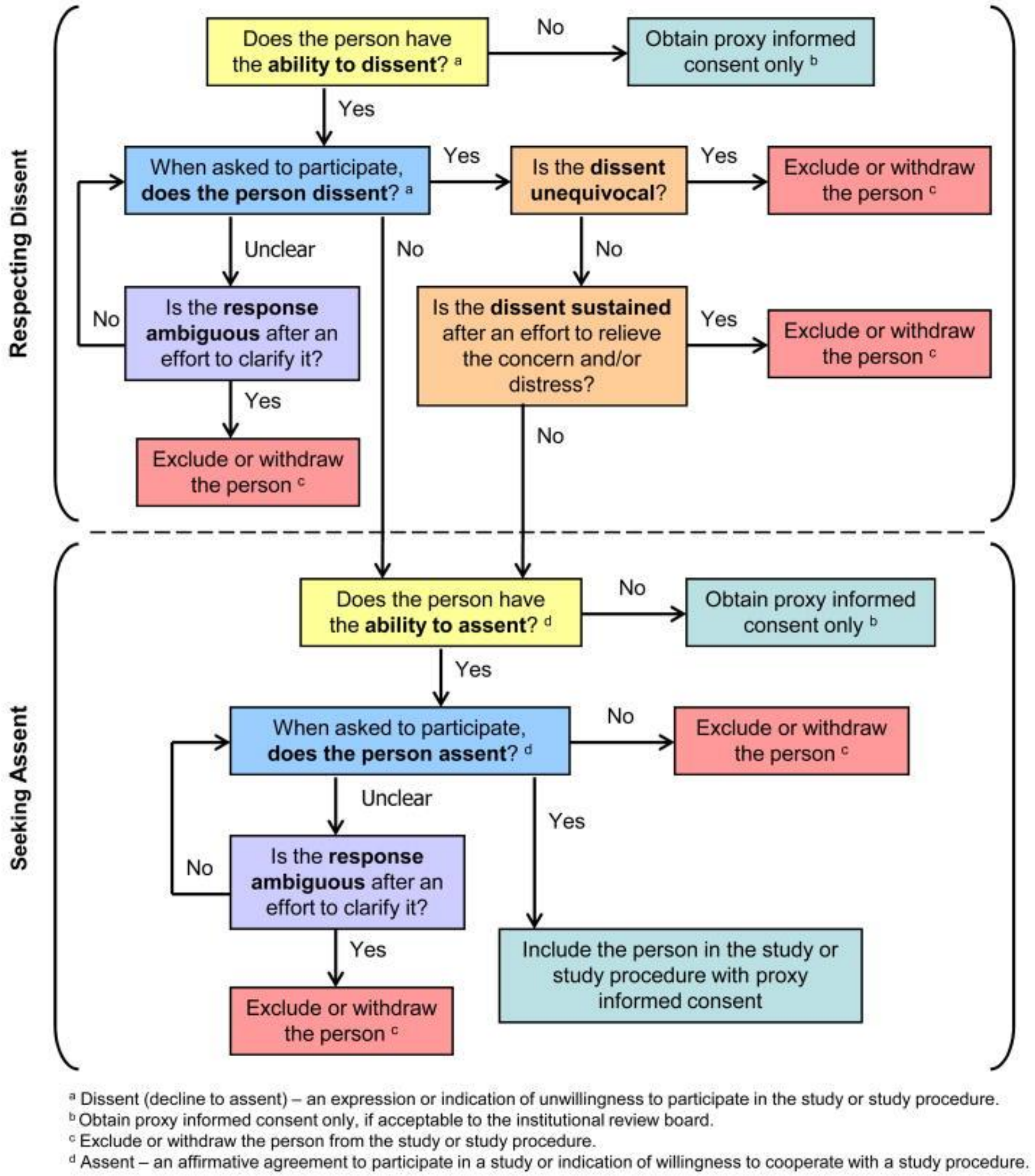

Figure 2. Decision Tree for Respecting Dissent and Seeking Assent for Dementia Research.
Source: Black et al. 2010 [35]

7.2 *Risk management and safety*

There are no obvious disadvantages or risks associated with the participation of subjects in this study. However, participants with cognitive impairment may have increased susceptibility to some forms of discomfort or distress. The research team have experience in working with people with cognitive impairment on previous research projects. Every effort will be taken to ensure these risks are mitigated as much as is possible. The research staff will be observant to the impact of information on the potential participant during interviews, providing appropriate rests, involving people familiar to the potential participant and ceasing any subjectively distressing interaction in a timely manner.

The potential risks will be reduced by the research team stressing that participation is strictly voluntary, and the participant can withdraw at any time without negative repercussions. The informed consent process will also highlight the nature of the study and the types of questions that will be asked.

If by taking part in this project, the participants suffer any injuries (including but not limited to physical or psychological distress) or complications as a result of this research project, the participants will be encouraged to contact the study team as soon as possible and they will be assisted with arranging appropriate treatment. If they are eligible for Medicare, they can receive any medical treatment required to treat the injury or complication, free of charge, as a public patient in any Australian public hospital. Any counselling or support will be provided by qualified staff who are not members of the research project team. This counselling will also be provided free of charge.

Participants may have existing relationships with their service provider organisations that have agreed to be study partners. For example, people with dementia may be recipients of interpreter services or dementia-related medical care, interpreters will be credentialed by the National Accreditation Authority for Translators & Interpreters (NAATI), and clinicians may have utilized interpreting services. The research team will be present for and monitor all contact between participants. Whether clients, family carers, clinicians, or interpreters take part in this research project, or not, should not have any bearing on their future relationship with their service provider, their employer, or accreditation body.

For interpreters, they will be assured that their consent is not coerced, that their participation is entirely voluntary and will not affect their relationship with their employer (Interpreter service) or the accreditation body (NAATI). Interpreters will be required to give informed consent before participating in the study and will be informed (both verbally and in writing) that they are free to withdraw from the study at any time.

For the co-design workshops, it is also possible that clinicians may be participants with interpreters they have worked with in the past. However, the research team are aware of the potential for unequal relationships and will not place familiar participants in the same co-design groups.

7.3 *Handling of withdrawals*

For participants who wish to withdraw after data has been collected, the data collected up to that time will form part of the research project results. Participants will be informed of this at the time of consent, on their PICFs.

7.3.1 People with dementia

Participants may withdraw from the interviews at any time. If they consent to participate but change their mind and do not wish to continue their involvement, the decision will be respected, and a replacement sought. People with dementia are not part of study 2 or 3.

7.3.2 Family carers

Participants may withdraw from the workshops at any time. If they consent to participate but change their mind and do not wish to continue their involvement, the decision will be respected, and a replacement sought. Carers are not part of study 2 or 3.

7.3.3 Interpreters

Participants are free with withdraw at any time from any of the three studies, with no effect on their relationships with the researchers, NAATI, or their employers. If they consent but then change their mind, the decision will be respected, and a replacement sought.

7.3.4 Clinicians

Participants are free with withdraw at any time from Study 1 and 3, with no effect on their relationships with the researchers or their employers. If they consent but then change their mind, the decision will be respected, and a replacement sought. Clinicians are not part of Study 2.

7.4 *Replacements*

7.4.1 Study 1

If there is time to organize a replacement, then the withdrawn participant will be replaced. If workshop participants withdraw on the day of the workshop, then no replacement will be made.

7.4.2 Study 2

If there is time to organise a replacement then the withdrawn participant will be replaced, up until the end of the first data collection point (baseline). As our study design requires 120 (60/60) participants to power study, our target of 150 (75/75) allows for some drop-out. However, if we anticipate that the target of 120 will not be reached, we may continue recruiting participants after the end of the first data collection point.

7.4.3 Study 3

If there is time to organize a replacement, then the withdrawn participant will be replaced.

8 Data Security and Handling

8.1 *Details of where records will be kept and how long will they be stored*

Any information obtained for the purpose of this research project that can identify participants will be treated as confidential and securely stored. It will be disclosed only with the participant's permission, or as permitted by law. Only members of the research team will have access to potentially identifying information. Industry partners or funding partners will only have access to de-identified aggregate project data.

The Project Lead Investigator A/Prof Brijnath has a conjoint position at Curtin University and NARI. As she is physically located at NARI in Melbourne, any physical copies of data will be kept in a locked filing cabinet on the NARI premises. Identifiers will be stored separately to individual data collection sheets. All digital data will be stored in a password-protected folder located on a secure file server, which is located at the NARI premises, and will also be securely stored on the Curtin University drives. A database of information will be created that will be used for this project only. The data will be kept securely at these locations for seven (7) years from the date of the last publication after which it will be destroyed.

8.2 *Confidentiality and security*

Participant confidentiality will be maintained along with the principles of good clinical practice guidelines (GCRP). No participant will be identifiable by name in any publications/presentations arising from the study. Data entry will be undertaken using a unique code number to identify participants. The code number key will be stored separately and only be accessible to the investigators of this study. Data collected will be entered directly into a secure electronic database and will be considered as source data. In any publication and/or presentation and data transferred overseas, information will be provided in such a way that participants cannot be identified. Hard copy data will be stored in locked filing cabinets at the National Ageing Research Institute. Electronic backups and all other electronic data will be stored in password protected electronic database in a deidentified format at Curtin University and NARI.

8.2.1 Study 1

Interviews and workshops will be recorded either using the Microsoft Teams inbuilt recording function or audio-recorders. Recordings will be transcribed by a professional transcription service, and then checked and de-identified by the research team. All recordings and transcripts will be stored electronically at NARI and Curtin University. If any notes taken by researchers during the workshops contain identifying information about participants, they will be stored in locked filing cabinet on NARI premises. Only de-identified data will be reported on.

8.2.2 Study 2

Participant details and covariates (age, sex, ethnicity, location, educational and economic attainments, and NAATI credential status) will be gathered directly from participants by the research team. Individual assessments scores will be undertaken at RMIT University and sent electronically to NARI, contained within password-protected zip files. The passwords for these zip files will be shared verbally. NARI researchers will enter individual scores into a database and with study ids assigned to each participant. NARI researchers will check data to ensure participants are not personally identifiable. This de-identified data will then be made available to other project partners for analysis and also saved on Curtin University drives.

8.2.3 Study 3

Data gathered by NAATI monitoring will gather aggregate data that is non-identifiable.

Interviews will be recorded either using the Microsoft Teams inbuilt recording function or audio-recorders. Recordings will be transcribed by a professional transcription service, and then checked and de-identified by NARI researchers. All recordings and transcripts will be stored electronically at NARI.

8.3 *Ancillary data*

Source data such as videos and photographs may be collected for promotional or training purposes and will be kept securely at NARI and Curtin University for 7 years from the date of publication. Participants will sign an informed consent form allowing us to use videos and photographs. If they choose not to have their images used, then researchers will not use this data.

Audio and video data will be stored on NARI's and Curtin's secure, password-protected server. Confidentiality of audio data will be assured as each audio file will be labelled with a unique code number (as outlined previously).

8.4 *Public availability of data*

Only aggregate and/or de-identified data will be publicly available on request. Only aggregate and/or de-identified data will be published.

9 References

[1] Guideline Adaptation Committee, Clinical Practice Guidelines and Principles of Care for People with Dementia, Sydney, 2016.

[2] G. Flores, The impact of medical interpreter services on the quality of health care: a systematic review, Med Care Res Rev 62(3) (2005) 255-99. http://doi.org/10.1177/1077558705275416

[3] B. Haralambous, J. Tinney, D. LoGiudice, S.M. Lee, X. Lin, Interpreter-mediated Cognitive Assessments: Who Wins and Who Loses?, Clin Gerontol 41(3) (2018) 227-236. http://doi.org/10.1080/07317115.2017.1398798

[4] R. Casas, E. Guzmán-Vélez, J. Cardona-Rodriguez, N. Rodriguez, G. Quiñones, B. Izaguirre, D. Tranel, Interpreter-Mediated Neuropsychological Testing of Monolingual Spanish Speakers, Clin Neuropsychol 26(1) (2012) 88-101. http://doi.org/10.1080/13854046.2011.640641

[5] C. Plejert, C. Lindholm, R.W. Schrauf, Multilingual Interaction and Dementia, in: C. Plejert, C. Lindholm, R.W. Schrauf (Eds.), Multilingual Interaction and Dementia, Multilingual Matters, Bristol, 2017, pp. 1-22.

[6] T.R. Nielsen, A. Vogel, M.W. Riepe, A. de Mendonca, G. Rodriguez, F. Nobili, A. Gade, G. Waldemar, Assessment of dementia in ethnic minority patients in Europe: a European Alzheimer's Disease Consortium survey, Int Psychogeriatr 23(1) (2011) 86-95. http://doi.org/10.1017/S1041610210000955

[7] A.R. Majlesi, C. Plejert, Embodiment in tests of cognitive functioning: A study of an interpreter-mediated dementia evaluation, Dementia 17(2) (2018) 138-163. http://doi.org/10.1177/1471301216635341

[8] C. Plejert, E. Antelius, M. Yazdanpanah, T.R. Nielsen, 'There's a letter called ef' on Challenges and Repair in Interpreter-Mediated Tests of Cognitive Functioning in Dementia Evaluations: A Case Study, J Cross Cult Gerontol 30(2) (2015) 163-87. http://doi.org/10.1007/s10823-015-9262-0

[9] A.R. Majlesi, E. Antelius, C. Plejert, Epistemic Negotiations in Interpreter-mediated Dementia Evaluations: The Cooperative Role of Patients' Relatives, in: C. Plejert, C. Lindholm, R.W. Schrauf (Eds.), Multilingual Interaction and Dementia, Multiligual Matters, Bristol, 2017, pp. 74-102.

[10] B. Haralambous, P. Mackell, X. Lin, M. Fearn, B. Dow, Improving health literacy about dementia among older Chinese and Vietnamese Australians, Aust Health Rev 42(1) (2018) 5-9. http://doi.org/10.1071/AH17056

[11] My Aged Care, My Aged Care Assessment Manual: For Regional Assessment Services and Aged Care Assessment Teams, Australian Government, Canberra, Australia, 2018. https://agedcare.health.gov.au/sites/default/files/documents/06_2018/my-aged-care-assessment-manual-june-2018-v1-1.pdf

[12] J. White, T. Plompen, C. Osadnik, L. Tao, E. Micallef, T. Haines, The experience of interpreter access and language discordant clinical encounters in Australian health care: a mixed methods exploration, Int J Equity Health 17(1) (2018) 151. http://doi.org/10.1186/s12939-018-0865-2

[13] A.S. Gilbert, S. Croy, K. Hwang, D. LoGiudice, B. Haralambous, Video remote interpreting for home-based cognitive assessments: stakeholders' perspectives Interpreting (In press) (2021).

[14] R.E. Glasgow, P.E. Estabrooks, Pragmatic Applications of RE-AIM for Health Care Initiatives in Community and Clinical Settings, Preventing chronic disease 15 (2018) E02. http://doi.org/10.5888/pcd15.170271

[15] J.S. Holtrop, B.A. Rabin, R.E. Glasgow, Qualitative approaches to use of the RE-AIM framework: rationale and methods, BMC health services research 18(1) (2018) 177. http://doi.org/10.1186/s12913-018-2938-8

[16] H. Arksey, L. O'Malley, Scoping studies: towards a methodological framework, International Journal of Social Research Methodology 8(1) (2005) 19-32. http://doi.org/10.1080/1364557032000119616

[17] E. Borgstrom, S. Barclay, Experience-based design, co-design and experience-based co-design in palliative and end-of-life care, BMJ Support Palliat Care 9(1) (2019) 60-66. http://doi.org/10.1136/bmjspcare-2016-001117

[18] J. Brown, D. Isaacs, The World Café : Shaping Our Futures Through Conversations That Matter, Berrett-Koehler Oakland, 2005.

[19] A. MacFarlane, R. Galvin, M. O'Sullivan, C. McInerney, E. Meagher, D. Burke, J.W. LeMaster, Participatory methods for research prioritization in primary care: an analysis of the World Cafe approach in Ireland and the USA, Fam Pract 34(3) (2017) 278-284. http://doi.org/10.1093/fampra/cmw104

[20] B.G. Dow, A., PITCH perfect for care workers, Australian Journal of Dementia Care 8(4) (2019) 12-13.

[21] B. Brijnath, J. Antoniades, J. Adams, C. Browning, D. Goeman, K. Ellis, M. Kent, Moving pictures: Raising awareness of dementia in CALD communities through multimedia, Innov Aging 3(Suppl 1) (2019) S452.

[22] NHMRC National Institute for Dementia Research (NNIDR), Culturally and linguistically diverse (CALD) dementia research action plan: Full report, NNIDR, Canberra, 2020.

[23] B. Dow, E. Gaffy, A.M.Y. Goh, C. Doyle, D. Ames, M. Winbolt, S. Savvas, S. Malta, P. Clarke, C. Cooper, A. Panayiotou, G. Livingston, C. Lyketsos, F. Batchelor, S.M. Loi, L.-F. Low, M. Polacsek, L. Gahan, J. Burton, B. Hallam, S. Scherer, F3-04-04: Co-designing a training programme for paid home carers to deliver dementia specific care to people at home, Alzheimers Dement 15(7S_Part_17) (2019) P869-P869. http://doi.org/10.1016/j.jalz.2019.06.4611

[24] N. Ono, T. Kiuchi, H. Ishikawa, Development and pilot testing of a novel education method for training medical interpreters, Patient Educ. Couns. 93(3) (2013) 604-611.

[25] D. Refki, M.P. Avery, D. Angela, Core Competencies for Healthcare Interpreters, Int J Humanit Soc Sci 3(2) (2013) 72-83.

[26] A.H. JØRgensen, Thinking-aloud in user interface design: a method promoting cognitive ergonomics, Ergonomics 33(4) (1990) 501-507. http://doi.org/10.1080/00140139008927157

[27] V. Venkatesh, M.G. Morris, G. Davis, F. Davis, User acceptance of information technology: Toward a unified view, MIS Quarterly 27(3) (2003) 425-478.

[28] I. Motola, L.A. Devine, H.S. Chung, J.E. Sullivan, S.B. Issenberg, Simulation in healthcare education: a best evidence practical guide. AMEE Guide No. 82, Med Teach 35(10) (2013) e1511-30. http://doi.org/10.3109/0142159x.2013.818632

[29] M. González-Davies, V. Enríquez-Raído, Situated learning in translator and interpreter training: bridging research and good practice, Interpret Transl Train 10(1) (2016) 1-11. http://doi.org/10.1080/1750399X.2016.1154339

[30] J. Hlavac, Mental Health Interpreting Guidelines for Interpreters Monash University, 2017. https://researchmgt.monash.edu/ws/portalfiles/portal/299061728/Mental_Health_Interpreting_Guidelines_for_Interpreters_29.XI.2017.pdf

[31] National Accreditation Authority for Translators and Interpreters (NAATI), Certified Interpreter Test Assessment Rubrics 2020. https://www.naati.com.au/wp-content/uploads/2020/10/Certified-Interpreter-Assessment-Rubrics.pdf

[32] National Accreditation Authority for Translators and Interpreters (NAATI), Intercultural Competency Test Rubrics, Canberra, 2020. https://www.naati.com.au/wp-content/uploads/2020/03/Intercultural-Competency-Test-Rubrics-Online.pdf

[33] B. Rosner, Fundamentals of biostatistics: Brooks, Cole, Cengage Learning (2011).

[34] WHO, Practical guidance for scaling up health service innovations, Geneva, 2009.

[35] B.S. Black, P.V. Rabins, J. Sugarman, J.H. Karlawish, Seeking assent and respecting dissent in dementia research, Am J Geriatr Psychiatry 18(1) (2010) 77-85. http://doi.org/10.1097/JGP.0b013e3181bd1de2
