# Supplementary material for: Interpreter Communication Quality in Cognitive Assessments for Dementia: The MINDSET Randomized Clinical Trial
Source: JAMA Netw Open. 2025 Feb 12;8(2):e2458069. doi: 10.1001/jamanetworkopen.2024.58069 (PMC11822547; doi:10.1001/jamanetworkopen.2024.58069)
Supplement: Supplement 2. — eTable 1. MINDSET Training Program Logic eTable 2. Number of Participants and Outcome Measures at Each Period eTable 3. Primary and Secondary Outcome Changes From Baseline, Main Analysis eTable 4. Primary and Secondary Outcome Changes From Baseline, Per-Protocol Analysis [file jamanetwopen-e2458069-s002.pdf]

Supplementary Online Content

Brijnath B, Markusevska S, Enticott J, et al. Interpreter communication quality in cognitive assessments for dementia: the MINDSET Randomized Clinical Trial. *JAMA Netw Open*. 2025;8(2):e2458069. doi:10.1001/jamanetworkopen.2024.58069

eTable 1. MINDSET Training Program Logic

eTable 2. Number of Participants and Outcome Measures at Each Period

eTable 3. Primary and Secondary Outcome Changes From Baseline, Main Analysis

eTable 4. Primary and Secondary Outcome Changes From Baseline, Per-Protocol Analysis

This supplementary material has been provided by the authors to give readers additional information about their work.

© 2025 Brijnath B et al. *JAMA Network Open*.

eTable 1: MINDSET Training Program logic

Interpreting domain	Learning outcomes	Assessment	Score weighting
1: Knowledge of dementia	Demonstrated knowledge of dementia, cognitive screening tools, dementia-related services, and person-centered care	DKAS—20 questions, 6 multiple-choice	15% DKAS, 5% multiple-choice questions
2: Cross-cultural awareness	Awareness of cross-cultural issues and ability to perform or mediate effective cross-cultural communication	3 Multiple-choice questions	10%
3: Briefings and debriefings	Understanding of preinteractional briefings, introductions, and postinteractional debriefings	1 Drag-and-drop checklist	10%
4: Interpreting skills	Demonstrated competency and accuracy interpreting clinicians' or assessors' speech, including assessment instructions; competency and accuracy interpreting patients' or clients' speech; effective interactional management skills	1 Video-simulated interpreting assessment	50%; hurdle requirement[a]
5: Ethical conduct	Demonstrated understanding of AUSIT ethical practice; demonstrated understanding of how to apply AUSIT ethical principles of interpreting into practice	2 Scenario-based questions	10%

Abbreviations: AUSIT, Australian Institute of Interpreters and Translators; DKAS, Dementia Knowledge Assessment Scale.
[a]If this item was not attempted, the participant's total assessment score was treated as missing data.

© 2025 Brijnath B et al. *JAMA Network Open*.

eTable 2: Number of participants and outcome measures at each period. Outcome data was missing due to incomplete data collection. The overall percentage of missing data is calculated using the denominator of 378 (=3*(62 + 64)). Missing data for primary outcome was 16.93% (64/378); domain 1, 12.43% (47/378); domain 2, 9.52% (36/378); domain 3, 9.52% (36/378); domain 4, 13.23% (50/378); and domain 5, 11.90% (45/378).

	**Control**	**Intervention**
**At Baseline**	**62**	**64**
Primary outcome	58	61
Domain 1: Knowledge of dementia	58	62
Domain 2: Cross-cultural awareness	62	64
Domain 3: Briefings/introductions	62	64
Domain 4: Interpreting skills	62	64
Domain 5: Ethical conduct	62	63
**At 3 months**	**55**	**56**
Primary outcome	49	47
Domain 1: Knowledge of dementia	51	56
Domain 2: Cross-cultural awareness	55	55
Domain 3: Briefings/introductions	55	55
Domain 4: Interpreting skills	52	49
Domain 5: Ethical conduct	53	51
**At 6months**	**54**	**52**
Primary outcome	50	49
Domain 1: Knowledge of dementia	53	51
Domain 2: Cross-cultural awareness	54	52
Domain 3: Briefings/introductions	54	52
Domain 4: Interpreting skills	51	50
Domain 5: Ethical conduct	53	51

© 2025 Brijnath B et al. *JAMA Network Open*.

eTable 3: Primary and secondary outcome changes from baseline (Main analysis). Intervention effects also shown.

	**3-month change from baseline**				**6-month change from baseline**			
	Control group	Intervention group	Intervention effect 95% CI	Intervention effect p-value	Control group	Intervention group	Intervention effect 95% CI	Intervention effect p-value
**Primary outcome**	1.6 ± 1.16	4.34 ± 1.03 †	2.74 (-0.43 to 5.9)	0.09	2.2 ± 0.95 *	3.71 ± 1.09 †	1.51 (-1.4 to 4.41)	0.31
**Secondary outcomes**								
Domain 1: Knowledge of dementia	0.49 ± 0.27	1.66 ± 0.25 †	1.16 (0.44 to 1.89) †	0.002	0.01 ± 0.3	1.15 ± 0.19 †	1.15 (0.43 to 1.87) †	0.002
Domain 2: Cross-cultural awareness	0.002 ± 0.22	0.5 ± 0.19 †	0.5 (-0.07 to 1.06)	0.08	0.08 ± 0.25	-0.08 ± 0.25	-0.16 (-0.84 to 0.52)	0.64
Domain 3: Briefings-debriefings introductions	0.54 ± 0.2 †	0.87 ± 0.21 †	0.33 (-0.25 to 0.91)	0.26	0.21 ± 0.16	0.79 ± 0.16 †	0.58 (0.12 to 1.04) *	0.01
Domain 4: Interpreting skills	1.32 ± 0.85	1.11 ± 0.73	-0.21 (-2.45 to 2.03)	0.85	2.47 ± 0.76 †	1.52 ± 1.04	-0.95 (-3.48 to 1.59)	0.46
Domain 5: Ethical conduct	0.24 ± 0.21	0.17 ± 0.19	-0.07 (-0.61 to 0.48)	0.81	-0.09 ± 0.22	-0.01 ± 0.2	0.08 (-0.49 to 0.64)	0.79

Data are means_± SEM or means (95% CI). Intervention effect between groups refers to the within-group change from baseline in the intervention group minus the within-group change from baseline in the control group † P < 0.01 within or between group difference from baseline; *P < 0.05 within or between-group difference from baseline.

© 2025 Brijnath B et al. *JAMA Network Open*.

eTable 4: Primary and secondary outcome changes from baseline (Per-protocol analysis). Intervention effects also shown.

	3-month change from baseline				6-month change from baseline			
	Control group	Intervention group	Intervention effect 95% CI	Intervention effect p-value	Control group	Intervention group	Intervention effect 95% CI	Intervention effect p-value
**Primary outcome**	1.32 ± 1.09	4.97 ± 1.09 †	3.65 (0.49 to 6.81) *	0.02	2.19 ± 0.83 †	4.06 ± 1.27 †	1.88 (-1.16 to 4.91)	0.23
**Secondary outcomes**								
Domain 1: Knowledge of dementia	0.68 ± 0.23 †	1.71 ± 0.3 †	1.03 (0.27 to 1.79) †	0.008	0.19 ± 0.26	1.17 ± 0.22 †	0.98 (0.31 to 1.65) †	0.004
Domain 2: Cross-cultural awareness	-0.04 ± 0.2	0.67 ± 0.2 †	0.71 (0.15 to 1.27) *	0.01	-0.08 ± 0.24	0.12 ± 0.27	0.2 (-0.52 to 0.92)	0.58
Domain 3: Briefings-debriefings introductions	0.42 ± 0.18 *	1.15 ± 0.22 †	0.73 (0.16 to 1.31) *	0.01	0.22 ± 0.14	0.93 ± 0.18 †	0.71 (0.24 to 1.18) †	0.003
Domain 4: Interpreting skills	1.15 ± 0.78	1.29 ± 0.82	0.14 (-2.14 to 2.42)	0.91	1.9 ± 0.82	2.12 ± 1.11	0.22 (-2.56 to 3.00)	0.87
Domain 5: Ethical conduct	0.15 ± 0.2	0.28 ± 0.16	0.14 (-0.36 to 0.63)	0.59	-0.07 ± 0.19	-0.03 ± 0.23	0.04 (-0.53 to 0.61)	0.89

Data are means_± SEM or means (95% CI). Intervention effect between groups refers to the within-group change from baseline in the intervention group minus the within-group change from baseline in the control group † P < 0.01 within or between group difference from baseline; *P < 0.05 within or between-group difference from baseline.

© 2025 Brijnath B et al. *JAMA Network Open*.
