# Supplementary material for: Interpreter Communication Quality in Cognitive Assessments for Dementia: The MINDSET Randomized Clinical Trial
Source: JAMA Netw Open. 2025 Feb 12;8(2):e2458069. doi: 10.1001/jamanetworkopen.2024.58069 (PMC11822547; doi:10.1001/jamanetworkopen.2024.58069)
Supplement: Supplement 3. — Data Sharing Statement [file jamanetwopen-e2458069-s003.pdf]

Data Sharing Statement

Brijnath. Interpreter Communication Quality In Cognitive Assessments for Dementia. *JAMA Netw Open*. Published February 12, 2025. doi:10.1001/jamanetworkopen.2024.58069

Data
Additional Information: Australian New Zealand Clinical Trials Registry,https://www.anzctr.org.au/, ACTRN12621001281886.
Data available: No

Additional Information
Explanation for why data not available: Only aggregate and/or de-identified data that support these findings will be made available upon request from the corresponding author. Access to the simulated videos and scripts used to assess Domain 4 (interpreter skills) are available from: https://osf.io/km5s8/
